# Cholesterol metabolism: from lipidomics to immunology

**DOI:** 10.1016/j.jlr.2021.100165

**Published:** 2021-12-22

**Authors:** William J. Griffiths, Yuqin Wang

**Affiliations:** Swansea University Medical School, Swansea, Wales, United Kingdom

**Keywords:** oxysterol, hydroxycholesterol, accessible cholesterol, macrophage, B cell, T cell, dendritic cell, virus, bacterial infection, membrane fusion, 3β,7α-diHCA, 3β,7α-dihydroxycholest-5-en-(25R)26-oic acid, 3β-HCA, 3β-hydroxycholest-5-en-(25R)26-oic acid, 7-DHC, 7-dehydrocholesterol, 7-OC, 7-oxocholesterol, 7α,25-diHC, 7α,25-dihydroxycholesterol, 7α,26-diHC, 7α,26-dihydroxycholesterol, 7β,25-diHC, 7β,25-dihydroxycholesterol, 7β,26-diHC, 7β,26-dihydroxycholesterol, 7α,25-diHCO, 7α,25-dihydroxycholest-4-en-3-one, 7α,26-diHCO, 7α,(25R)26-dihydroxycholest-4-en-3-one, 7α-HC, 7α-hydroxycholesterol, 7β-HC, 7β-hydroxycholesterol, 20S-HC, 20S-hydroxycholesterol, 22R-HC, 22R-hydroxycholesterol, 24S-HC, 24S-hydroxycholesterol, 24S,25-EC, 24S,25-epoxycholesterol, 25-HC, 25-hydroxycholesterol, 26-HC, (25R)26-hydroxycholesterol, ACE2, angiotensin-converting enzyme 2, AID, activation-induced cytidine deaminase, AIM2, absent in melanoma 2, ALO, anthrolysin O, APO, apolipoprotein, BCG, Bacillus Calmette-Guérin, BMDM, bone marrow-derived macrophage, CDC, cholesterol-dependent cytolysin, CESD, cholesterol ester storage disease, *Ch25h*, cholesterol 25-hydroxylase, CNS, central nervous system, COVID-19, coronavirus disease 2019, CSF, cerebrospinal fluid, CYP, cytochrome P450, DC, dendritic cell, DDA, dendrogenin A, DDAB, didodecyldimethylammonium bromide, DHCR7, dehydrocholesterol reductase 7, EAE, experimental autoimmune encephalitis, EBI2, Epstein-Barr virus-induced gene 2, EBP, emopamil binding protein, HEK293T, human embryonic kidney 293T cells, HIV, human immunodeficiency virus, HMGCR, HMG-CoA reductase, HSD, hydroxysteroid dehydrogenase, HSV-1, herpes simplex virus 1, ICZ, itraconazole, IFNAR, IFNα receptor, IgA, immunoglobulin A, IL-2, interleukin 2, INSIG, insulin-induced gene, JAK, Janus kinase, KDO, Kdo2-lipid A, LBD, ligand binding domain, LIPA, lysosomal acid lipase A, LPS, lipopolysaccharide, LXR, liver X receptor, mCMV, mouse cytomegalovirus, mTORC1, mammalian target of rapamycin complex 1, MyD88, myeloid differentiation primary response protein 88, NIH, National Institutes of Health, NLR, nucleotide binding domain and leucine-rich repeat, NLRP3, NOD-, LRR, and pyrin domain-containing protein 3, NPC, Niemann-Pick C, nSREBP-2, the nuclear form of SREBP-2, PFO, perfringolysin O, ROR, retinoic acid-related orphan receptor, ROS, reactive oxygen species, SARS-CoV, severe acute respiratory syndrome coronavirus, SCAP, SREBP cleavage-activating protein, SOAT1, sterol O-acyltransferase 1, SPG5, spastic paraplegia type 5, STAT1, signal transducer and activator of transcription protein 1, TGF-β1, transforming growth factor beta 1, Th, T helper, TLR3, Toll-like receptor 3, TLR4, Toll-like receptor 4, TMPRSS2, transmembrane protease/serine subfamily member 2, TRIF, Toll/IL-1R domain-containing adaptor-inducing IFNβ, VSV, vesicular stomatitis Indiana virus

## Abstract

Oxysterols, the oxidized forms of cholesterol or of its precursors, are formed in the first steps of cholesterol metabolism. Oxysterols have interested chemists, biologists, and physicians for many decades, but their exact biological relevance in vivo, other than as intermediates in bile acid biosynthesis, has long been debated. However, in the first quarter of this century, a role for side-chain oxysterols and their C-7 oxidized metabolites has been convincingly established in the immune system. 25-Hydroxycholesterol has been shown to be synthesized by macrophages in response to the activation of Toll-like receptors and to offer protection against microbial pathogens, whereas 7α,25-dihydroxycholesterol has been shown to act as a chemoattractant to lymphocytes expressing the G protein-coupled receptor Epstein-Barr virus-induced gene 2 and to be important in coordinating the action of B cells, T cells, and dendritic cells in secondary lymphoid tissue. There is a growing body of evidence that not only these two oxysterols but also many of their isomers are of importance to the proper function of the immune system. Here, we review recent findings related to the roles of oxysterols in immunology.

## Graphical abstract

**Figure:**
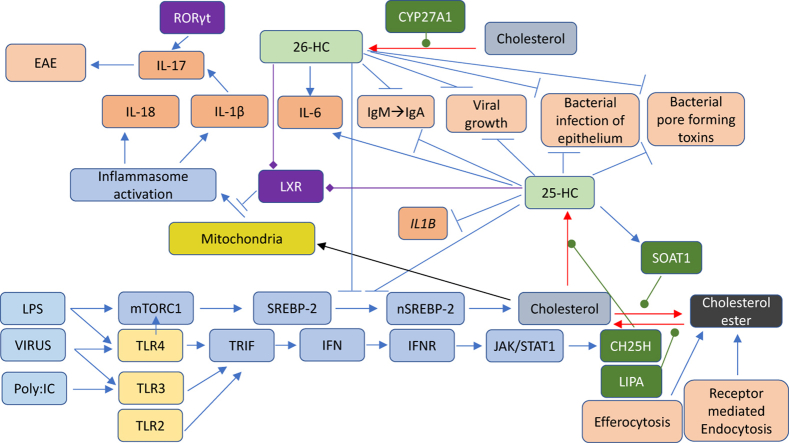

Lipidomic studies performed in the last 15 years have revolutionized our understanding of the relationship between cholesterol metabolism and the immune system. In the years 2009–2010, Dennis *et al.* (1) reported the biosynthesis of 25-hydroxycholesterol (25-HC, see supplemental data for chemical structures) in macrophages in response to stimulation by Kdo_2_-lipid A (KDO), the active component of lipopolysaccharide (LPS), also known as endotoxin, found on the outer membrane of Gram-negative bacteria, which acts as a Toll-like receptor 4 (TLR4) agonist (Fig. 1); Diczfalusy *et al.* (2) found that cholesterol 25-hydroxylase (*Ch25h*) was strongly upregulated by LPS and that injection of LPS into healthy volunteers increased their plasma 25-HC; whereas Bauman *et al.* (3) found that treatment of naive B cells with 25-HC (nanomolar) suppressed interleukin 2 (IL-2)-mediated stimulation of B-cell proliferation, repressed activation-induced cytidine deaminase (AID) expression, and suppressed immunoglobulin A (IgA) class switching in B cells. These results demonstrated a mechanism for the negative regulation of the adaptive immune system by the innate immune system in response to bacterial infection (3). Not only is 25-HC generated by macrophages in response to TLR4 ligands but also Park and Scott (4) showed that *Ch25h* is upregulated in dendritic cells (DCs), antigen-presenting cells of the immune system, in response to cell surface TLR4 activation by LPS and to intracellular Toll-like receptor 3 (TLR3) ligands. Park and Scott uncovered the pathway for *Ch25h* regulation to be via the production of type I IFNs and signaling through the IFNα receptor (IFNAR) and the Janus kinase (JAK)/signal transducer and activator of transcription protein 1 (STAT1) pathway. One year later in 2011, studies by Hannedouche *et al.* (5) and Liu *et al.* (6) identified 7α,25-dihydroxycholesterol (7α,25-diHC) as a chemoattractant for immune cells expressing the G protein-coupled receptor, GPR183, also known as Epstein-Barr virus-induced gene 2 (EBI2). They showed that 7α,25-diHC is required to position activated B cells within the spleen to the outer regions of follicles, and its absence leads to reduced plasma cell response after immune challenge demonstrating a role for 7α,25-diHC in the adaptive immune response (Fig. 2A) (5). See Ref. (7) for an excellent description of the link between GPR183 and B cells.

**Fig. 1:**
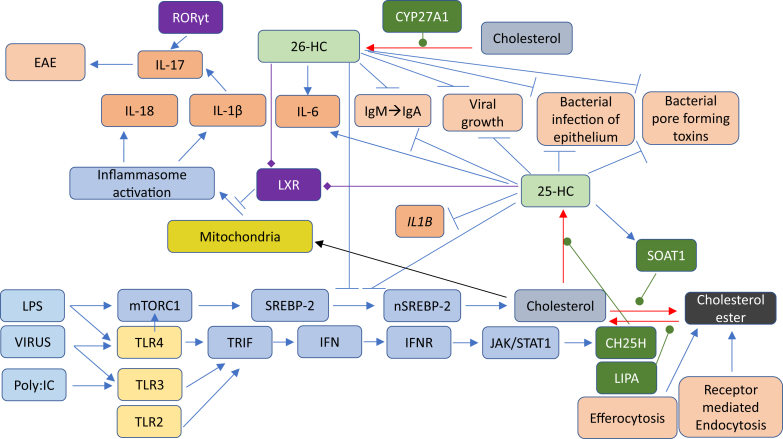
Pathway map summarizing the involvement of 25-HC in the immune response. The involvement of 26-HC in some of the pathways is also shown. Blue arrows signify a “process,” red arrows a chemical reaction, Τ signifies inhibition of a process, black arrows indicate transport, arrows with a diamond arrowhead indicate activation of a receptor, and green oval arrowheads indicate catalysis. Oxysterols are on a light green background, enzymes are on a dark green background, nuclear receptors are on a purple background, ILs on a dark salmon background, and end processes on a light salmon background. See supplemental data for oxysterol structures.

**Fig. 2:**
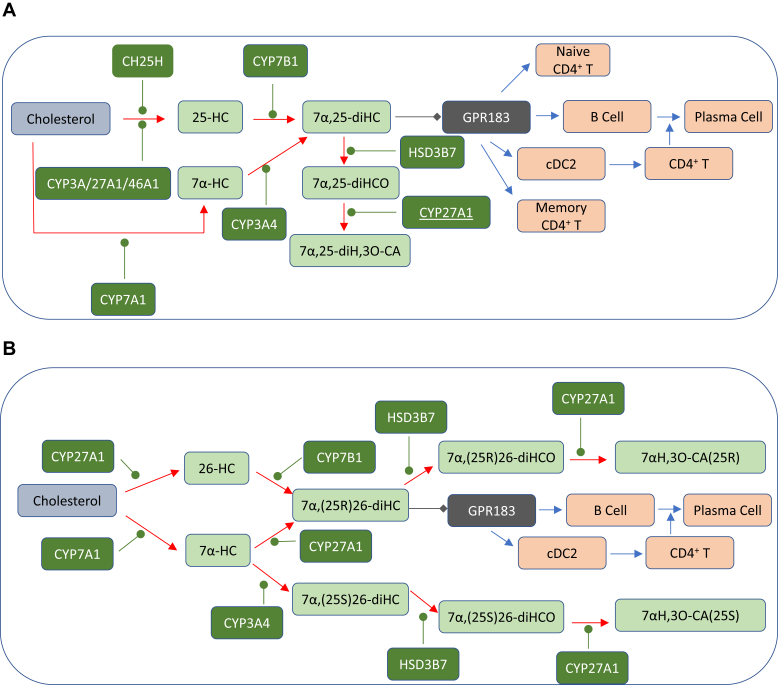
Pathway map detailing the biosynthesis and metabolism of (A) 7α,25-diHC and (B) 7α,(25R)26-diHC and 7α,(25S)26-diHC. 7α,25-diHC and 7α,(25R)26-diHC are ligands toward GPR183 (shown on a *dark gray* background) as indicated by a diamond arrowhead; other symbols are as in Fig. 1. Proposed but unproven enzymes are underlined. Abbreviations for sterols are given in the text.

Dennis *et al*. (1), Diczfalusy *et al*. (2), Bauman *et al.* (3), and Park and Scott (4) demonstrated the enhanced expression of CH25H in immune cells in response to bacterial infection, whereas two articles published in 2013 further established *Ch25h* as an IFN-stimulated gene and its product 25-HC to be antiviral against a broad range of enveloped viruses (8, 9). In combination, these two studies indicate that 25-HC blocks membrane fusion between the virus and the host cell, ultimately inhibiting viral growth. The exact mechanism is likely to vary from virus to virus and cell to cell, but there is good evidence that it involves inhibition of the processing of SREBP-2 to its active form as a transcription factor for genes of the cholesterol biosynthesis pathway and uptake (9, 10). More recently, the replication of both severe acute respiratory syndrome coronavirus (SARS-CoV) and SARS-CoV-2 (coronavirus disease 2019 [COVID-19]) has been found to be suppressed by 25-HC (11, 12, 13, 14). The suppression of viral replication is likely to be at viral entry with 25-HC repressing membrane fusion between the viral envelope and the lipid bilayers of the cells (13, 14).

## Side-chain oxysterols

25-HC is one of the best studied cholesterol metabolites; this is on account of its commercial availability and low cost. Unlike most other primary cholesterol metabolites, it is not biosynthesized from cholesterol by a cytochrome P450 (CYP) enzyme but by an enzyme, CH25H, that utilizes a diiron cofactor to catalyze hydroxylation (15), although some CYP enzymes also have capacity to introduce a hydroxy group to the sterol side chain at C-25 in a side reaction to their main activity (16, 17, 18, 19, 20). In contrast to its formation, the metabolism of 25-HC has been less well studied, although 7α-hydroxylation by CYP7B1 to give 7α,25-diHC followed by oxidation by hydroxysteroid dehydrogenase (HSD) 3B7 to give 7α,25-dihydroxycholest-4-en-3-one (7α,25-diHCO) is a well-established route (Fig. 2A) (21, 22). It is likely that 7α,25-diHCO is further metabolized to the acid, 7α,25-dihydroxy-3-oxocholest-4-en-26-oic acid (23).

**Fig. 3:**
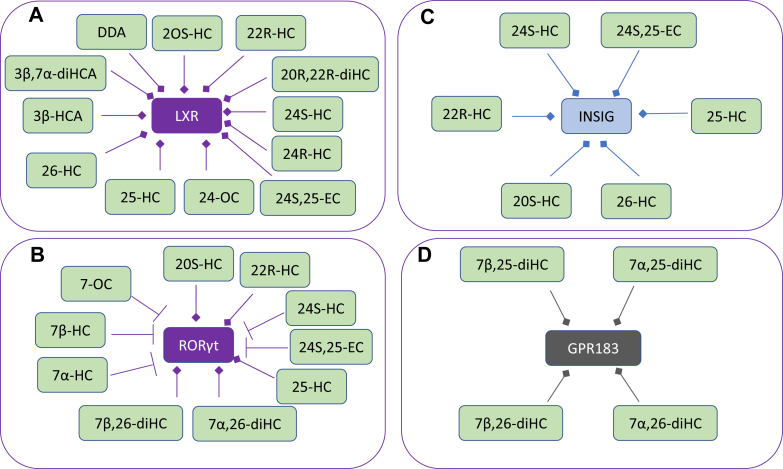
Oxysterols act as ligands to (A) LXRs, (B) RORγ, (C) INSIG, and (D) GPR183. Ligand interactions are indicated by a diamond arrowhead, whereas a T signifies inverse agonists.

**Fig. 4:**
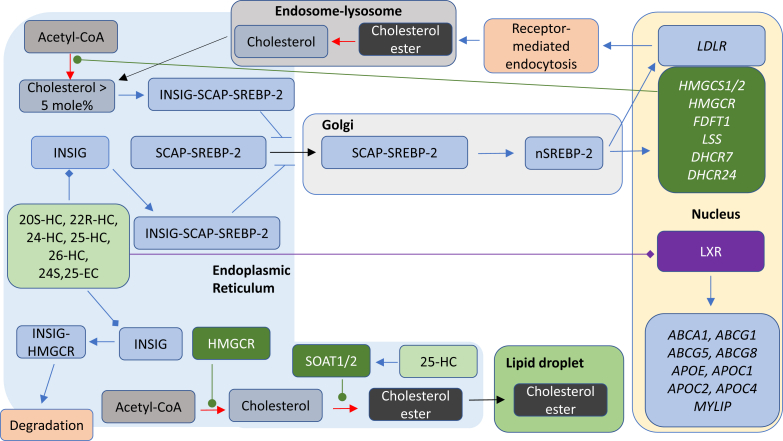
Summary of the mechanisms of cholesterol uptake, synthesis, and storage and the involvement of oxysterols and of cholesterol in cholesterol regulation. The cell can take up cholesterol esters via lipoprotein receptors, or cholesterol can be synthesized via the mevalonate pathway under SREBP-2 regulation. Excess cholesterol can be stored as cholesterol esters in lipid droplets or exported by ABC transporters onto lipoprotein particles. Symbols are as indicated in Figs. 1 and 2. Cell compartments are color coded, endoplasmic reticulum light blue, Golgi light gray, endosome-lysosomal compartment gray, and nucleus pale yellow.

25-HC is a ligand to the liver X receptors (LXRα, NR1H3; LXRβ, NR1H2) (24, 25, 26). LXR target genes include *i*) *ABC* transporters that are important for cholesterol absorption from the intestine via ABCA1-mediated efflux of cholesterol from enterocytes into the circulation, excretion into the intestine via ABCG5/8-mediated efflux from enterocytes into the intestinal lumen, and for cholesterol efflux from cells in general through ABCA1; *ii*) apolipoproteins (*APO*s) coding for APOE, APOC1, APOC2, and APOC4 proteins that transport cholesterol between cells, and *iii*) *IDOL* (inducible degrader of the LDL receptor or *MYLIP,* myosin regulatory light chain-interacting protein), which is important in regulating the uptake of cholesterol by cells (27). LXRs are involved in the inflammatory response and in the regulation of the lipid composition of membranes (27). Besides 25-HC, other side-chain oxysterols similarly activate LXRs, including 24S,25-epoxycholesterol (24S,25-EC), 20S-hydroxycholesterol (20S-HC), 22R-hydroxycholesterol (22R-HC), 20R,22R-dihydroxycholesterol, 24S-hydroxycholesterol (24S-HC), 24R-hydroxycholesterol, 24-oxocholesterol (24-OC, also known as 24-ketocholesterol), (25R)26-hydroxycholesterol (26-HC, also more commonly known by the nonsystematic name 27-hydroxycholesterol (28)), 3β-hydroxycholest-5-en-(25R)26-oic acid (3β-HCA), and 3β,7α-dihydroxycholest-5-en-(25R)26-oic acid (3β,7α-diHCA; Fig. 3A) (24, 26, 29, 30, 31). To avoid unnecessary confusion, the reader can assume in this article that unless stated otherwise, products of C-26 oxidation have 25R stereochemistry. It should be noted that some ring-substituted oxysterols also activate LXRs, for example, dendrogenin A (DDA) (32). Besides being an agonist toward LXRs, 25-HC and many related oxysterols inhibit the processing of SREBPs (33, 34). SREBP-1c and SREBP-2 are the major forms of SREBP found in liver, SREBP-1c mainly regulates the expression of enzymes involved in the synthesis of fatty acids, whereas SREBP-2 preferentially regulates the expression of cholesterol biosynthetic genes (35). SREBP-2 is synthesized in the endoplasmic reticulum where it binds to the transport protein SREBP cleavage-activating protein (SCAP). The function of SCAP is to transport SREBP-2 to the Golgi for processing to its active form as a transcription factor (the nuclear form of SREBP-2 [nSREBP-2]; Fig. 4). Insulin-induced gene (INSIG) is an endoplasmic reticulum-resident protein, and 25-HC will bind to INSIG causing a conformational change, which results in the binding of INSIG to SCAP and anchoring of the INSIG-SCAP-SREBP-2 complex in the endoplasmic reticulum, thereby preventing SREBP-2 processing to its active form (34). 25-HC will also interact with INSIG to activate the proteolysis of HMG-CoA reductase (HMGCR) (33, 36). Cholesterol will also regulate its own synthesis via binding to SCAP, causing a conformational change that will result in SCAP binding to INSIG and similarly anchoring the INSIG-SCAP-SREBP-2 complex in the endoplasmic reticulum (33). While cholesterol is the main regulator of its own synthesis, 25-HC and other oxysterols can have acute effects on synthesis.

There is considerable crosstalk between the LXR and SREBP-2 pathways. Activation of the former by oxysterols will lead to elimination of excess cholesterol from cells via ABCA1-mediated export and reverse cholesterol transport, whereas inhibition of the latter will lead to reduced cholesterol synthesis and uptake. The net result will be a reduction in cellular cholesterol.

## Bacterial infection, LPS, and oxysterols

### Lipidomics

As mentioned previously, LPS acts as a TLR4 agonist. LC-MS lipidomic analysis by Dennis *et al.* (1) of the mouse macrophage RAW264.7 activated by KDO, the active component of LPS, revealed a 3-fold increase in intracellular 25-HC accompanying a 4-fold increase in *Ch25h* mRNA (37). Over the 24 h time course of stimulation, the cellular cholesterol level doubled as did that of 24S,25-EC (1). The increase in 24S,25-EC is likely to reflect the increased synthesis of cholesterol as the two are generated in parallel pathways (38). The increase in cellular cholesterol and 24S,25-EC is likely to involve LPS stimulation of mammalian target of rapamycin complex 1 (mTORC1)-driven activation of the SREBP-2 pathway, as discussed later (Fig. 1) (39, 40). In a related study, Baumann *et al.* (3) found a 100-fold and 200-fold increase in *Ch25h* mRNA in intraperitoneal macrophages and bone marrow-derived macrophages (BMDMs), respectively, when challenged with KDO. CH25H protein was also elevated, and 25-HC levels secreted into the medium rose to about 65 nM (26 ng/ml) and 500 nM (200 ng/ml) by the peritoneal macrophages and BMDM, respectively, from almost zero (3). Note, no saponification step was performed; hence, these concentrations are for the unesterified sterol. Confusion can arise when sterol concentrations are given and, in this review, values given are for the free unesterified molecule unless stated otherwise. Agonists toward TLR2 and TLR3 were similarly found to stimulate *Ch25h* expression in intraperitoneal macrophages, and intraperitoneal injection of KDO to mice confirmed the in vivo induction of *Ch25h* mRNA with the consequent elevation of 25-HC levels in tissues, whereas incubation of macrophages with LPS derived from *Escherichia coli* and *Salmonella enterica* confirmed that bacterial LPS induces *Ch25h* expression (3).

What might be the function of 25-HC synthesized by macrophages in response to TLR activation? A clue comes from the increased serum IgA levels in the *Ch25h*^−/−^ mouse (3). In vitro experiments showed that treatment of naive B cells with 25-HC (nanomolar) blocked class-switch recombination, suppressing IgA secretion. Class-switch recombination is a process by which B cells change Ig production from one type to another, and as part of the adaptive immune response, B cells switch from producing immunoglobulin M to synthesizing and secreting IgA. The switch can be mediated in a T cell-dependent or T cell-independent mechanism. 25-HC was found to inhibit both T cell-dependent (cytokine mediated) and T cell-independent class-switch recombination. IC_50_ (half-maximal inhibitory concentration) for 25-HC-mediated suppression of class-switch recombination was found to be about 50 nM (20 ng/ml), a concentration less than that found in the media of KDO-activated macrophages (3). Interestingly, 26-HC suppressed IgA production after cytokine stimulation of B cells, but other LXR agonists 24S-HC and 22R-HC did not. Dihydrolanosterol, an intermediate in the Kandustch-Russell pathway of cholesterol biosynthesis, and an inhibitor of cholesterol biosynthesis by blocking both the processing of SREBP-2 to its active form and accelerating the INSIG-induced degradation of HMGCR (41), was also inactive in the B-cell assay (3). These results suggest that the suppressive effect of 25-HC toward class-switch recombination is independent of total cellular cholesterol levels.

The mechanism by which 25-HC inhibits IgA production in B cells via macrophage stimulation by LPS in combination with the cytokines IL-5 and transforming growth factor beta 1 (TGF-β1) was found to involve AID, a deaminase involved in the initiation of class-switch recombination. 25-HC blocks the induction of *AID* mRNA in response to a combination of LPS, IL-5, and TGF-β1 but not by LPS alone (3). A second mechanism by which 25-HC, and also 26-HC, inhibits IgA production in B cells is through reducing proliferation of B cells in response to IL-2 but not to TGF-β1 and IL-5 (3). 25-HC also inhibits class-switch recombination in a T-cell-independent manner, as evidenced by inhibiting the effect of APRIL (a proliferation-inducing ligand), which is released by DCs. Importantly, *Ch25h*^−/−^ mice have high levels of IgA in sera, mucosa, and lungs, whereas *Cyp7b1*^−/−^ mice where 25-HC is high have low levels of IgA in sera, mucosa, and lungs (3). In summary, the study of Bauman *et al.* (3) showed that activation of the innate immune system through macrophage TLRs induces CH25H and the production of 25-HC. 25-HC then acts as a suppressor of class-switch recombination in B cells to negatively regulate the adaptive immune system.

At about the same time that Bauman *et al.* (3) were investigating the effects of LPS on mouse macrophages, Diczfalusy *et al.* (2) found that intravenous injection of LPS into humans increased plasma 25-HC levels, measured as total 25-HC following saponification of sterol esters, doubling about 4 h after injection. During these 4 h, there was no increase in 26-HC. The study performed by Diczfalusy *et al.* was aimed initially at identifying genes upregulated by LPS in mouse BMDM. Similar to Dennis *et al.* (1), Diczfalusy *et al.* (2) and Bauman *et al*. (3) found that LPS, and also live *E. coli*, increased the expression of *Ch25h* in macrophages. As identified by Bauman *et al*., the increase in *Ch25h* was transitory, reaching a maximum at about 4 h and returning to normal by 12 h after treatment. The involvement of TLR4 in *Ch25h* induction was confirmed in mice deficient in this gene where induction of *Ch25h* was severely impaired (2). Importantly, there was an increase neither in the expression of *Cyp27a1* (2), the gene coding the enzyme CYP27A1, which can also produce 25-HC as a byproduct to its major product 26-HC (20, 42) nor *Cyp7b1* coding CYP7B1, which converts 25-HC to 7α,25-diHC (21). In agreement with Bauman *et al*. (2), there was a significant increase in 25-HC production in response to LPS 6 h after treatment, which was maintained at 24 h.

In 2010, Park and Scott (4) made the important discovery that *Ch25h* production in macrophages and DCs is regulated by type I IFNs and that *Ch25h* is an IFN-responsive gene. They found that besides upregulating the expression of *Ch25h*, agonists of intracellular TLR3 and cell surface TLR4 also induced *Ifnb1* transcription and proposed a pathway in which activation by TLR3 (poly I:C) and TLR4 (LPS) ligands induces *Ch25h* through TRIF (Toll/IL-1R domain-containing adaptor-inducing IFNβ)- mediated production of type I IFNs and signaling through the IFNAR and the JAK/STAT1 pathway (Fig. 1). They also found that the type II IFN, IFNγ, upregulated *Ch25h* also through STAT1 (4). As signaling events initiated by TLRs rely on the adaptor proteins, myeloid differentiation primary response protein 88 (MyD88) and TRIF, Park and Scott made use of *Myd88*^−/−^ and *Trif*^−/−^ mice to investigate *Ch25h* induction. TLR3 and TLR4 agonists failed to activate *Ch25h* transcription in bone marrow-derived DCs from *Trif*^−/−^ mice, whereas in DCs from *Myd88*^−/−^ mice, the expression of *Ch25h* was no different, or only modestly reduced, compared with controls treated with TLR3 and TLR4 agonists. These results suggest that TLR-mediated expression of *Ch25h* is primarily through a TRIF-mediated rather than a MyD88-mediated mechanism (4). These data agreed with that from Diczfalusy *et al.* (2) who found that BMDM from the *Myd88*^−/−^ mouse responded toward LPS in a similar manner to controls.

In combination, the studies of Dennis *et al*. (1), Diczfalusy *et al.* (2), Bauman *et al*. (3), and Park and Scott (4) indicate that the pathway leading to CH25H expression and 25-HC production in macrophages and DCs consists of TLR3/4 → TRIF → IFN regulatory factor 3/NF-κB → IFNβ → IFNAR → JAK/STAT1 → CH25H → 25-HC (43).

The immune function of CH25H was soon confirmed by Zou *et al.* (44) in a study of *Listeria monocytogenes* infection, whereas Liu *et al.* (45) showed *CH25H* to be an IFN-stimulated gene with antiviral activity. Although the study by Bauman *et al.* (3) indicated class-switch recombination to be LXR independent, Tontonoz *et al.* (46, 47) have shown that inflammatory signaling induced by LPS through TLR4 can regulate the expression of LXR target genes, and LXR activation can negatively regulate the expression of inflammatory genes. With respect to the innate immune system, Ecker *et al.* (48) have shown that 25-HC can suppress the differentiation of monocytes to macrophages. Perhaps, providing a negative feedback mechanism to reduce the number of macrophages generating 25-HC (43).

## Oxysterols, cholesterol, and accessible cholesterol

25-HC and other side-chain oxysterols can regulate cellular cholesterol through activating LXR receptors (24, 25, 26), by interacting with INSIG and repressing SREBP-2 processing (34), accelerating the degradation of the HMGCR (36, 49) and by stimulating the endoplasmic reticulum enzyme acyl-CoA:cholesterol acyl transferase, also known as sterol O-acyltransferase 1 (SOAT1), to esterify cholesterol (Fig. 4) (50). Such activities are used by cells to regulate or manipulate their plasma membrane cholesterol, the major location of cellular cholesterol. 25-HC and other oxysterols cross membranes at a much faster rate than cholesterol (51, 52, 53), and for hydroxycholesterols, there appears to be a correlation between the separation of the added hydroxy group from the 3β-hydroxy group and the rate of membrane transit (26 > 24S > 4β) (52). This suggests that side-chain oxysterols could have a paracrine activity or an autocrine activity in modulating plasma membrane cholesterol levels.

**Fig. 5:**
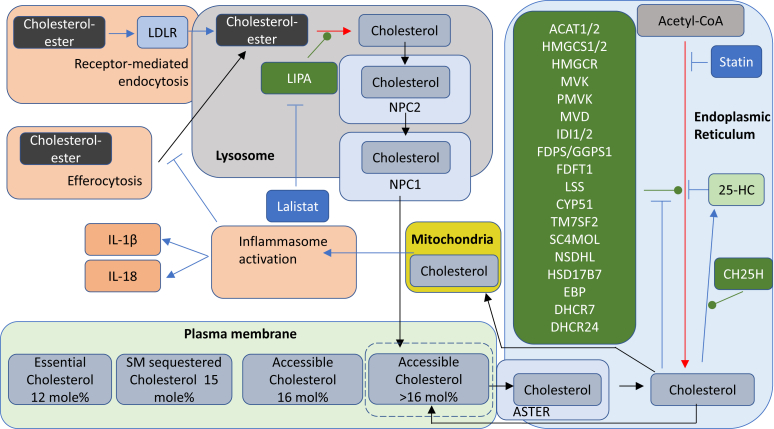
The role of accessible cholesterol in regulating cholesterol synthesis and the role of cholesterol in inflammasome activation. Pharmacological mediators are on a royal blue background, other symbols, and cell compartments are as used in earlier figures. Plasma membrane is in light green, and mitochondria is in mustard.

The plasma membrane contains between 60% and 90% of a cells total cholesterol (54, 55), and plasma membrane cholesterol may make up 40–50 mol % of plasma membrane lipids (54, 56); however, INSIG, SCAP, SREBP-2, HMGCR, and SOAT1 are all located in the endoplasmic reticulum, an organelle that contains less than 1% of the cell's cholesterol (57) and where cholesterol only makes up 5 mol % of the membrane lipids (58). This leads to the question, how might a machinery in the endoplasmic reticulum of a cell sense the cholesterol level of a cell when the majority of its cholesterol is located in the plasma membrane? To answer this question, it is important to remember that under homeostatic conditions, cellular cholesterol regulates its own synthesis and import (33). When the cholesterol level in the endoplasmic reticulum exceeds 5 mol % of total lipids, cholesterol binds to SCAP, causing a conformational change that results in SCAP binding to INSIG and tethering the INSIG-SCAP-SREBP-2 complex in endoplasmic reticulum, thereby preventing processing of SREBP-2 in the Golgi to its active form (nSREBP-2) as the master regulator of cholesterol biosynthesis and of the expression of the LDL receptor (Fig. 4) (33). To explain how cholesterol in the plasma membrane can regulate cholesterol biosynthesis and uptake through the machinery located in the endoplasmic reticulum, Das *et al.* (59) in 2014 introduced the concept of three pools of plasma membrane cholesterol. They proposed that in the plasma membrane, there are three pools of cholesterol: *i*) a pool of cholesterol that is “accessible” to receptor proteins and to transport to the endoplasmic reticulum, *ii*) a SM sequestered pool that can be released by sphingomyelinase, and *iii*) a residual pool of cholesterol essential for plasma membrane integrity. The three pools in cholesterol-replete cells correspond to about 16, 15, and 12 mol % of total plasma membrane lipids, respectively (59). When cholesterol in the plasma membrane is in excess following LDL uptake by receptor-mediated endocytosis, ester hydrolysis by lysosomal acid lipase A (LIPA), and transport by Niemann-Pick C (NPC)2 and NPC1 proteins to the lysosome outer leaflet and ultimately by transfer to the plasma membrane, the result is a rise in accessible cholesterol, which is then transported to the endoplasmic reticulum to switch off cholesterol synthesis and expression of the LDL receptor (Fig. 5) (59). Conversely, statin treatment results in inhibition of cholesterol synthesis, concomitant plasma membrane accessible cholesterol depletion, an overall fall in endoplasmic reticulum cholesterol levels, and switching back on the SREBP-2 pathway. The key to this regulatory process is accessible cholesterol, which through transfer to the endoplasmic reticulum via ASTER (GRAM domain containing 1, GRAMD1) proteins (60, 61, 62), switches the SREBP-2 pathway off and on (63).

The idea of accessible cholesterol is derived from studies performed by Lange and Steck (64, 65) in Illinois and Radhakrishnan and McConnell (66, 67) in Stanford. Accessible cholesterol can be considered as a descriptive term for cholesterol molecules with a high thermodynamic chemical activity (68). Chemical activity being related to the environment of a compound; in this case, cholesterol in a phospholipid bilayer. Cholesterol complexed with phospholipids (sphingolipids and glycerophospholipids) is in a lower energy environment and has lower chemical activity than uncomplexed free cholesterol, which is in a higher energy environment, and accessible to transport from the plasma membrane (59, 66). In a membrane, cholesterol exists in stoichiometric complexes with sphingolipids, glycerophospholipids, and proteins, and when membrane cholesterol exceeds the binding capacity of these membrane components, this free cholesterol has a high thermodynamic chemical activity and becomes accessible cholesterol (65).

Accessible cholesterol is measured through its availability to protein probes, for example, bacterial cholesterol oxidase enzymes (69), its extraction by cyclodextrins, for example, methyl-β-cyclodextrin (66), and by its binding to bacterial pore-forming toxins, that is, the cholesterol-dependent cytolysins (CDCs), perfringolysin O (PFO) and anthrolysin O (ALO) (70, 71). A mutant form of PFO, PFO∗, binds to accessible cholesterol but does not form pores at 4°C and is widely exploited to probe for accessible cholesterol (59, 63, 70), whereas a subdomain of ALO, ALOD4, will bind to accessible cholesterol but not form pores (71, 72). All these methods provide a readout for cholesterol accessibility against increasing cholesterol concentration that is sigmoidal or J shaped, that is, there is little change in cellular accessible cholesterol as cholesterol concentration is increased in the membrane until a threshold or equivalence point is reached, at which point cholesterol accessibility rises sharply (65, 68).

Side-chain oxysterols can be imagined as an autocrine form or a paracrine form of accessible cholesterol. Like accessible cholesterol, they will provide a signal to repress SREBP-2 processing, but they will cross membranes much faster than cholesterol, so may be likened to “fast-acting” accessible cholesterol.

## Oxysterols, pore-forming toxins, and bacterial transmission

In 2020, two articles appeared, both of which showed 25-HC to be protective against bacterial infection by depleting accessible cholesterol (73, 74, 75). These findings were supported by a third article published in 2021 demonstrating 26-HC to have a similar bioactivity (76).

### CDCs

As discussed previously, modified forms of CDCs, that is, bacterial pore-forming toxins, can be used to monitor accessible cholesterol; however, the native forms induce pore formation and ultimately cell death. However, Zhou *et al.* (73) have shown that macrophages and neutrophiles can reprogram their sterol metabolism to provide resistance to CDC pore formation. CDCs bind to cholesterol in the plasma membrane of the target cell, oligomerize, and create pores resulting in loss of membrane integrity and ultimately cell death. Zhou *et al.* (73) discovered that PFO activation of TLR3 provides protection against pore formation. As discussed previously, activation of TLR3 in macrophages leads to IFNβ formation, and both IFNβ and IFNγ were found to induce resistance to PFO. IFNβ and IFNγ also provided resistance against the pore-forming toxins streptolysin O and ALO. Both IFNs also protected neutrophils against PFO. Both IFNβ and IFNγ treatments of macrophages decreased ALOD4 binding (a measure of accessible cholesterol); this linked the protective effects of IFNs to a reduction of plasma membrane accessible cholesterol, although no decrease was found in overall cell cholesterol (73).

What might be the link between activation of TLR3, IFN, accessible cholesterol, and resistance against CDC pore-forming toxins? As *Ch25h* is an IFN-stimulated gene (4, 43), 25-HC, the product of the translated enzyme, might provide such a link. Zhou *et al.* (73) found that macrophages when treated with 25-HC (3 μM, 1.2 μg/ml, 4 h) showed reduced ALOD4 binding, but when *Ch25h*^−/−^ macrophages were treated with IFNs, ALOD4 binding was not reduced, and *Ch25h*^−/−^ macrophages were not protected against PFO or streptolysin O challenge. However, when 25-HC itself was added to *Ch25h*^−/−^ or control macrophages, they were protected from PFO challenge. In addition, simvastatin reduced ALOD4 binding to otherwise unstimulated macrophages and protected against CDC-mediated membrane damage. As 25-HC is derived from cholesterol, in combination, these results led to the proposal that endogenous production of cholesterol and its metabolism is involved in macrophage susceptibility to, and protection against, CDCs (73).

**Fig. 6:**
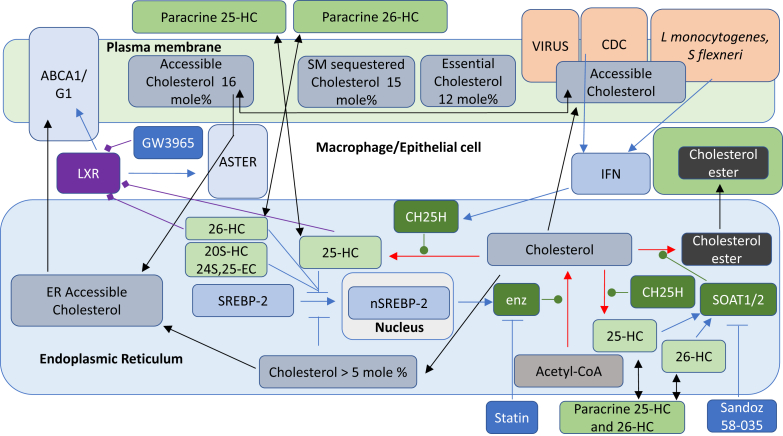
Oxysterols, accessible cholesterol, and protection against microbial infection. Symbols and color coding are as in earlier figures. enz corresponds to enzymes of the cholesterol biosynthesis pathway.

25-HC can reduce cholesterol biosynthesis and uptake by inhibiting SREBP-2 processing (34), it can activate LXRs and enhance cholesterol export (26, 27, 77), and mediate cholesterol ester formation (50). Using ^13^C-isotope tracer studies, IFNβ stimulation of control macrophages led to a drastic fall in cholesterol synthesis; in contrast, in *Ch25h*^−/−^ macrophages, there was only a small although significant attenuation in cholesterol synthesis. Although IFN treatment of *Ch25h*^−/−^ does not attenuate ALDO4 binding, addition of simvastatin does, supporting the concept that IFN-mediated protection against CDC is via 25-HC inhibition of cholesterol biosynthesis (Fig. 6). *Abca1* and *Abcg1* are LXR target genes and code for cholesterol efflux transporters (27). However, *Abca1*^−/−^ macrophages are no more susceptible to PFO than control macrophages, and IFN treatment provided similar protection to both genotypes. In addition, ALOD4 binding was found to be similarly reduced in control, *Abca1*^−/−^, and *Abcg1*^−/−^ macrophages upon IFN treatment (73). This led to the conclusion that under the experimental conditions employed, protection against PFO conveyed by IFNβ and IFNγ in mouse BMDM was not dependent on cholesterol efflux. However, the synthetic LXR agonist GW3965 provided protection to control and *Ch25h*^−/−^ macrophages against PFO challenge and also against ALOD4 binding (a measure of accessible cholesterol), implying that LXR activation might have a support role in protection against CDCs (73).

Included among the IFN-stimulated genes expressed by macrophages are *Soat1* and *Soat2*. Surprisingly, however, the presence of cholesterol in cell media was not required for increased cholesterol esterification in macrophages in response to IFNs. This suggests that the source of cholesterol for esterification is from host cell membranes (73) or directly from synthesis in the endoplasmic reticulum. Zhou *et al.* (73) went on to provide strong evidence that cholesterol ester formation contributes to IFN-mediated resistance to CDCs in macrophages by showing that a pharmacological inhibitor (Sandoz 58-035) of SOAT1 and SOAT2 attenuates protection provided by IFNs against PFO.

Zhou *et al.* (73) were able to confirm the protective effect of 25-HC against infection by CDC in vivo. They found that *Ch25h*^−/−^ mice were more susceptible to infection by CDC injected in skin than WT animals, but preinjection of 25-HC reduced subsequent tissue damage.

In combination, the data of Zhou *et al.* favor a model where IFNs via 25-HC reduce cholesterol synthesis and increase esterification of cholesterol, resulting in a reduction of accessible cholesterol required for CDC binding (Fig. 6). This mechanism is exploited by immune cells to evade pathogens and protect the host from damage and is likely to be exploited by cells other than macrophages including those of epithelial tissue.

### Epithelial cell infection

In fact, soon after the publication of the data by Zhou *et al.*, Ormsby *et al.* (76) showed that bovine endometrial epithelial cells generate 25-HC in response to challenge by LPS and pyolysin, the CDC from *Trueperella pyogenes*, a bacterium that targets endometrial cells. Ormsby *et al.* (76) found that 25-HC protected endometrial epithelial and stromal cells against pyolysin and also against α-hemolysin, a CDC from *Staphylococcus aureus*. The endometrium is the innermost lining layer of the uterus, and to investigate further the potential source of cytoprotective oxysterols, Ormsby *et al.* profiled using LC-MS, the oxysterol content of epithelial cells, stromal cells, and also uterine and ovarian follicular fluids. Even without bacterial challenge, epithelial cells were found to secrete 25-HC (about 1 ng/10^5^ cells) and also 7α,25-diHCO at about the same level. Following pyolysin challenge, these values more than doubled. Stromal cells were not found to secrete either oxysterol before or after pyolysin challenge. Uterine and follicular fluids collected from cattle with no evidence of infection were found to contain not only 25-HC (3.9 ± 0.6 ng/ml uterine, 26.8 ± 11.4 ng/ml follicular) and its metabolite 7α,25-diHC (1.3 ± 0.7 ng/ml uterine, 2.0 ± 1.5 ng/ml follicular) but also high levels of 26-HC (19.9 ± 4.9 ng/ml uterine, 244.6 ± 99.5 ng/ml follicular) and its metabolites 3β-HCA (12.7 ± 6.8 ng/ml uterine, 295.8 ± 118.4 ng/ml follicular), 3β,7α-diHCA (4.9 ± 2.5 ng/ml uterine, 6.5 ± 4.2 ng/ml follicular), and 7α-hydroxy-3-oxocholest-4-en-(25R/S)26-oic acid (81.3 ng/ml uterine, 258.9 ± 136.8 ng/ml follicular). 26-HC, like 25-HC, was found to be cytoprotective toward epithelial and stromal cells against both pyolysin and α-hemolysin, although unlike 25-HC, 26-HC was not released by endometrial cells in response to pathogen challenge (76). Based on studies using the SOAT inhibitor Sandoz 58-035, knockdown of LXRα and LXRβ, and measurement of plasma membrane accessible cholesterol binding to pyolysin, Ormsby *et al.* (76) concluded that cytoprotection against pyolysin by 26-HC, and by inference 25-HC, was by activation of LXRs and SOAT resulting in a reduction in plasma membrane accessible cholesterol and thereby binding of CDCs to the plasma membrane. Importantly, the concentrations of 25-HC and 26-HC required for cytoprotection of endometrial cells, 5 and 25 ng/ml, respectively, were similar to concentrations of these oxysterols found in uterine fluid, lending weight to a hypothesis that oxysterols provide protection against pathogens in the uterus. Two further points of consideration are that besides 25-HC and 26-HC, 3β-HCA and 3β,7α-diHCA are LXR ligands (24, 29, 30, 31) and are present in uterine fluid and could potentially offer additional protection against CDCs via LXR activation, perhaps through an additive effect, whereas Griffin *et al.* (78) have shown that statins and knockdown of *HMGCR* partially protect bovine endometrial cells against polylysin implicating inhibition of the SREBP-2 pathway in protection against CDCs.

IFNγ is an important component of the mucosal epithelium immune system, which provides a barrier to bacteria protecting underlying tissue, and deficiency in IFNγ increases host susceptibility to *L. monocytogenes* and *Mycobacterium tuberculosis* (79). In their recent study, Abrams *et al.* (74) exploited the *L. monocytogenes* to investigate how IFNγ-activated macrophages protect the barrier epithelium from bacterial infection. *L. monocytogenes* penetrates mucosal membranes of the gut, spreads to other organs, and it is often used as a model enteric pathogen to study the immune response to bacterial infection (74). IFNγ-stimulated macrophages secrete 25-HC in response to induction of *Ch25h* (9), and Abrams *et al.* (74) found that conditioned media containing elevated levels of 25-HC suppressed *L. monocytogenes* infection of epithelial cells. Importantly, conditioned media from *Ch25h*^−/−^ macrophage cultures, where 25-HC is absent, failed to suppress *L. monocytogenes* infection of epithelia cells. These data led to the suggestion that 25-HC is the active molecule secreted by IFNγ-activated macrophages that inhibits *L. monocytogenes* infection of epithelial cells. This hypothesis was confirmed by further in vitro and in vivo experiments where 25-HC added to culture media suppressed bacterial colonization of a wide range of immortalized cells, and when 25-HC was injected into mice infected with *L. monocytogenes*, the bacterial burden was reduced (74). Importantly, and in agreement with earlier studies (44), 25-HC did not protect BMDM from *L. monocytogenes* infection (74). However, the antibacterial activity of 25-HC in epithelial cells against *L. monocytogenes* was explained by suppression of contact-dependent cell-to-cell spread across host cells; this could be reconciled with 25-HC depleting plasma membrane accessible cholesterol (Fig. 6) (74). Although Abrams *et al.* (74) did not find a reduction in total cholesterol in 25-HC-treated cells (CHO cells), accessible cholesterol in the plasma membrane was reduced as revealed by reduced ALOD4 binding. *Shigella flexneri* is a Gram-negative bacterium and infects the intestine, and like *L. monocytogenes*, its intercellular dissemination is inhibited by reducing accessible cholesterol.

To uncover the mechanism by which 25-HC reduces accessible cholesterol, Abrams *et al.* (74) performed investigations on CHO-K1 cells, a subclone of the original CHO cell line. They found that besides 25-HC, 20S-HC and 26-HC also reduce accessible cholesterol in these cells as determined by ALOD4 binding. These three oxysterols will inhibit the processing of SREBP-2 to its active form and thereby downregulate cholesterol biosynthesis and uptake (Fig. 4) (34, 74). 22R-HC will also suppress SREBP-2 processing but surprisingly did not reduce ALOD4 binding in CHO-K1 cells (74). 22R-HC will also activate the LXR receptors (25), arguing that neither reducing cholesterol biosynthesis nor enhancing cholesterol efflux via activation of LXRs provide a general mechanism for side-chain oxysterol mobilization of accessible cholesterol in CHO-K1 cells.

As discussed previously, 25-HC enhances the activity of SOAT, the endoplasmic reticulum enzymes that catalyze the formation of cholesterol esters (50). Interestingly, the SOAT-specific inhibitor, Sandoz 58-035, prevented the elimination of accessible cholesterol stimulated by 25-HC, 20S-HC, and 26-HC and also suppressed the antibacterial activity of 25-HC in CHO-7 cells. These data point to cholesterol ester formation, to be the primary driver for plasma membrane remodeling induced by 25-HC, although this may in addition be sustained by inhibition of SREBP-2 processing (Fig. 6) (74). Perhaps surprisingly in light of the activity of 25-HC in protecting macrophages against pore-forming toxins produced by bacteria, 25-HC was found to make macrophages more susceptible to *L. monocytogenes* infection (44, 74).

In summary, Abrams *et al.* found that in response to IFNγ epithelia, cells are protected against both *L. monocytogenes* and *S. flexneri*. Protection is by way of mobilizing accessible cholesterol away from the plasma membrane and enhancing its esterification by 25-HC-mediated activation of SOAT and on a longer time scale by 25-HC inhibiting new cholesterol synthesis and uptake preventing the replenishment of membrane accessible cholesterol (Fig. 6).

## Anti-inflammatory activity of 25-HC

### Inflammasomes and IL-1β

Considering that *Ch25h* is an IFN-stimulated gene (4, 9, 43) and that its metabolic product, 25-HC, has multiple activities that regulate cellular sterols (Fig. 4) (27, 33, 50) raises the question: are the known suppressive effects of type I IFNs on the immune system (80, 81) mediated by 25-HC and its regulation of sterols?

Type I IFNs are known to downregulate inflammasome activity and IL-1β production (82), IL-1β being a cytokine produced by activated macrophages as a proprotein, which is cleaved by caspase-1 to the mature inflammatory mediator, whereas inflammasomes are multiprotein complexes that function to activate caspase-1 for its proteolytic action. Inflammasome activation is triggered by a sensor protein from the nucleotide binding domain and leucine-rich repeat (NLR) family, from the absent in melanoma 2 (AIM2) (absent in melanoma-like receptor) family or by pyrin protein (Mediterranean fever innate immunity regulator). Upon ligand binding, inflammasome sensor proteins oligomerize, recruit the adaptor protein ASC (apoptosis-associated speck-like adaptor protein containing a C-terminal caspase recruitment domain), which oligomerizes and recruit procaspase-1, which undergoes autoproteolysis to release active caspase-1, which can proteolyze pro-IL-1-family cytokines (IL-1β and IL-18) to their active forms (83).

In a groundbreaking study, Reboldi *et al.* (84) showed that *Ch25h*^−/−^ macrophages overproduce IL-1 family cytokines in response to LPS; this increase is transient peaking after 8 h of stimulation. IL-1 cytokine overproduction was explained by 25-HC having an inhibitory effect on *Il1b* expression and on the activation of IL-1β protein by inflammasomes. A mechanism was proposed whereby bacterial infection, or LPS treatment, of macrophages leads to upregulation of IFN expression and CH25H-mediated 25-HC formation (Fig. 1). 25-HC represses SREBP-2 processing and expression of enzymes of the cholesterol biosynthesis pathway, which ultimately leads to downregulation of inflammasome-mediated proteolysis of pro-IL-1β to its active form; 25-HC also represses *Il1b* expression (84). The overall effect being suppressed inflammation. A downside of suppressed inflammation is reduced resistance against bacterial infection, and Reboldi *et al.* (84) showed that *Ch25h*^−/−^ mice have less bacterial growth in spleen and liver than *Ch25h*^*+/−*^ littermate controls following infection by *L. monocytogenes*. *Ch25h*^−/−^ mice also showed elevated IL-1β and IL-18 in serum and BMDM cultures. These data are in agreement with earlier data of Zou *et al.* who showed that 25-HC failed to protect BMDM from *L. monocytogenes* infection (44) and the later report by Abrams *et al.* showing that 25-HC makes macrophages more susceptible to *L. monocytogenes* infection (74). Interestingly, Reboldi *et al.* (84) found *Ch25h*^−/−^ mice to have elevated IL-17A^+^ T cells in spleen and lymph nodes and also an increased neutrophil count; these cell populations often promote inflammation. An increase in IL-17A^+^ T cells is likely to be mediated by elevated IL-1β, one of the cytokines involved in the differentiation of T helper (T_h_) 17 cells. Experimental autoimmune encephalitis (EAE) is a mouse model of multiple sclerosis driven by IL-17 (80), and Reboldi *et al.* (84) found that *Ch25h*^−/−^ mice show exacerbated EAE compared with controls. Thus, 25-HC is likely to be protective against EAE by limiting IL-1β and IL-17A^+^ cell populations (Fig. 1). The conclusion of this study was that 25-HC acts as a mediator in a negative-feedback pathway of IFN on IL-1 family cytokine production and inflammasome activity (84).

In a follow-on study, Dang *et al.* (85) discovered how 25-HC prevents inflammasome activation. They uncovered a link between mTORC1 stimulation by LPS, expression of SREBP-2 target genes, and cholesterol over accumulation in *Ch25h*^−/−^ macrophages and proposed that 25-HC functions to restrain cholesterol biosynthesis and thereby inhibit inflammasome activation and IL-1β secretion (Fig. 1) (85). After 8 h stimulation with LPS, *Ch25h*^−/−^ macrophages were found by LC-MS to have elevated levels of desmosterol, 7-dehydrocholesterol (7-DHC), and lanosterol compared with control cells, suggesting that both the Bloch and Kandustch-Russell pathways of cholesterol biosynthesis are repressed by 25-HC. Unsurprisingly in light of these data, cholesterol levels in *Ch25h*^−/−^ macrophages were elevated after 8 h stimulation. Interestingly, at 4 h of LPS stimulation, macrophages of both genotypes showed a decrease in their cholesterol content; this is consistent with *Ch25h* transcripts peaking at 4 h (2, 3) and transcripts of HMG-CoA synthase 1 (*Hmgcs1*), squalene synthase (*Sqs*, farnesyl-diphosphate farnesyltransferase 1, *Fdft1*), lanosterol synthase (*Lss*), and dehydrocholesterol reductase 24 (*Dhcr24*) being repressed at 2 h in both *Ch25h*^−/−^ and control macrophages. However, the mRNAs had increased after 8 h of LPS stimulation in *Ch25h*^−/−^ macrophages. In summary, these data indicate that 25-HC is required by macrophages following 8 h of LPS stimulation to repress mTORC1-induced activation of the SREBP-2 pathway and to avoid overaccumulation of cholesterol (85).

Dang *et al.* (85) also showed that it is the AIM2 inflammasome that is involved in cholesterol-dependent IL-1β secretion in response to LPS or *L. monocytogenes* infection of macrophages. AIM2 is a DNA sensor protein, and Dang *et al.* (85) discovered that cholesterol overload of mitochondria can lead to mitochondrial DNA release into the cytosol and AIM2-inflammasone activation leading to proteolysis of pro-IL-1β to the active cytokine. In summary, the work of Reboldi *et al.* (84) and Dang *et al.* (85) revealed a mechanism by which 25-HC protects macrophages from overstimulation of the immune system. The pathway involves LPS activation of the TLR receptors leading to mTORC1 signaling, which in the absence of 25-HC leads to enhanced expression of SREBP-2 target genes and cholesterol synthesis resulting in mitochondrial cholesterol overload and AIM2-inflammasome activation with subsequent enhanced IL-1β secretion (Fig. 1). TLR activation will also mediate IFN production and induction of IFN-stimulated genes, which include *Ch25h*. In turn, this will lead to the production of 25-HC, which will repress the processing of SREBP-2 and restrain mTORC1-mediated overproduction of cholesterol, thereby preventing AIM2-inflammasome activation, limiting the magnitude of the inflammatory response (Fig. 1). It is likely that NLR pyrin domain-containing protein 3 (NLRP3) inflammasomes, which are activated by a diverse array of stimuli (86), including cholesterol crystals (87), also contribute redundantly to IL-1β production, perhaps via oxidized mitochondrial DNA (85). It is also possible that activation of LXR may have a role in restraining the pathological secretion of IL-1β, as the synthetic LXR agonist GW3965 was found to be protective against mitochondrial dysfunction in macrophages overexpressing *Hmgcr* or *Dhcr24*, although 25-HC still blocked IL-1β release in *LXRα*^−/−^β^−/−^ macrophages, arguing against a dominant role for LXR in restraining IL-1β secretion (84, 85). 25-HC can enhance the removal of free cholesterol by activating SOAT1 and converting it to cholesterol esters; this could also be a mechanism to protect against cholesterol overload in macrophages. Although the studies of Reboldi *et al.* (84) and Dang *et al.* (85) focused on the pathological consequence of LPS stimulation of macrophages in a setting of infection (84, 85), cholesterol overload may have a role in driving in chronic diseases, such as obesity, metabolic syndrome, and atherosclerosis (83).

### Efferocytosis, LIPA, and 25-HC

Efferocytosis is the process by which macrophages phagocytose billions of host cells per day, preventing inflammatory consequences of apoptotic debris. It is in this context that Viaud *et al.* (88) showed 25-HC to have a role in protecting macrophages against mitochondrial-induced NLRP3 inflammasome activation and defective cell clearance. A consequence of ingestion of apoptotic cells is the import of large amounts of cholesterol esters to the macrophage. These are hydrolyzed within the lysosome by the enzyme LIPA to free cholesterol and fatty acids. One of the metabolic routes for released cholesterol is conversion to an oxysterol. Using LC-MS, Viaud *et al.* found that 3 h post efferocytosis, THP-1-derived macrophages treated with the LIPA inhibitor lalistat had greatly reduced levels of 25-HC, 26-HC and 4β-hydroxycholesterol (4β-HC), 70%, 50%, and 50% reduced respectively, compared with controls (Fig. 5). These changes in oxysterol abundance were a consequence as reduced substrate availability as opposed to reduced expression of mRNA of the respective biosynthetic genes *Ch25h*, *Cyp27a1*, and *Cyp3a4* (88). LIPA inhibition led to reduced efferocytotic capacity of macrophages, NLRP3 inflammasome activation, and higher IL-1β and IL-18 secretions. In control macrophages, ingestion of apoptotic cells leads to downregulation of *Srebf-2* (the gene coding SREBP-2) and *Hmgcr* 3 h after efferocytosis, effects absent in lalistat-treated macrophages but restored by addition of 25-HC. Addition of 25-HC reduced inflammasome activation after efferocytosis in lalistat-treated macrophages and restored an efficient efferocytic response. This led to the conclusion that inhibition of LIPA results in reduced 25-HC formation, accentuated cholesterol synthesis, mitochondrial-induced inflammasome activation, and reduced clearance of apoptotic cells (Fig. 5). While 25-HC has an effect on inflammasome activation within 3 h of efferocytosis, surprisingly, 26-HC does not prevent inflammasome activation, but acting as an LXR ligand can partially restore efferocytic capacity of lalistat-treated macrophages. Viaud *et al.* (88) showed that reduced LXR activation in lalistat-treated macrophages impaired cholesterol efflux and reduced efferocytic capacity. The study by Viaud *et al.* (88) provides a link between cholesterol hydrolysis, LIPA, oxysterols, macrophage efferocytic capacity, and metabolic inflammation. These results imply that it is cholesterol newly synthesized in the endoplasmic reticulum that is responsible for mitochondrial-induced inflammasome activation rather than cholesterol released by LIPA, and that hydrolysis of cholesterol esters will provide a source of 25-HC, and presumably cholesterol, to inhibit the biosynthesis of new cholesterol. A possible explanation for the actual source of cholesterol affecting mitochondrial-induced inflammasome activation comes from its availability for transport to the mitochondria. The data of Viaud *et al.* suggest that newly synthesized cholesterol is in a more accessible environment than that passing through the lysosome, which first needs to be transported to the plasma membrane before reaching the endoplasmic reticulum and mitochondria (Fig. 5).

In human, LIPA deficiency results in two major phenotypes of cholesterol ester storage disease (CESD; Mendelian Inheritance in Man, MIM, number: 278000): infant-onset Wolman disease, where there is no or <1% of normal LIPA activity, and late-onset CESD. Infants with Wolman disease often do not survive beyond 12 months of life. Late-onset CESD is often undiagnosed and can present in infancy, childhood, or in adults. CESD presents with hepatomegaly, splenomegaly, dyslipidemia, and accelerated atherosclerosis (89). Patients with Wolman's disease often present with an inflammatory phenotype, including hemophagocytic lymphohistiocytosis, where the body makes too many activated immune cells. A new treatment introduced by Potter *et al.* (89) in Royal Manchester Children's Hospital in the United Kingdom is enzyme replacement therapy with dietary substrate reduction followed by hematopoietic stem cell transplant. As of 2021, of the five patients treated in Manchester in this manner, four are still alive more than 40 months after multimodal treatment, and both phenotype and laboratory parameters are improved compared with treatment with enzyme replacement therapy alone; histologically, there are reduced cholesterol clefts and fewer foamy macrophages (89). Cholestane-3β,5α,6β-triol is a marker of presentation and the worsening of Wolman's disease (90); it is likely to be formed by cholesterol epoxide hydrolase (a dimer of 7-DHC reductase [DHCR7] and Δ^8^-Δ^7^ isomerase and emopamil binding protein sterol isomerase [EBP]), two enzymes of the cholesterol biosynthesis pathway, catalyzed hydration of 3β-hydroxycholestane-5,6-epoxide (5,6-epoxycholesterol) (91), a nonenzymatic product of cholesterol (92, 93), potentially formed in mitochondria under oxidative stress in Wolman's disease. After multimodal treatment, all five patients showed greatly reduced plasma cholestane-3β,5α,6β-triol as measured by LC-MS (89). Two of the five patients had presented before treatment with hemophagocytic lymphohistiocytosis, which is reported in many cases of Wolman's disease, and is suggested to involve inflammasome activation (94). This provides a link between the clinical work of Potter *et al.* (89) on patients with defective LIPA, the in vivo experiments of Viaud *et al.* (88) using the LIPA inhibitor lalistat, which resulted in inflammasome activation of macrophages, and reduced efferocytic capacity, which can be corrected by addition of 25-HC (88, 89). It is not unreasonable to speculate that inflammasome activation in Wolman's disease is a consequence of mitochondrial cholesterol overload by newly synthesized cholesterol (Fig. 5).

Using LC-MS, we have performed a sterolomic profile of Wolman's disease patients from the Royal Manchester Children’s Hospital prior to stem cell transplantation as part of a larger study of unusual oxysterols produced in lysosomal storage disorders (95). At the time of this study, we were unaware of the work of Viaud *et al.* (88) and were surprised to find an absence of 25-HC in plasma from Wolman's disease patients (95). Plasma levels of this oxysterol are usually low at 0.5–2 ng/ml (nonesterified) in healthy individuals as are those of cholestane-3β,5α,6β-triol (0.5–5 ng/ml, nonesterified); however, as reported by Potter *et al.* (89), levels of the latter oxysterol are elevated in plasma from Wolman's disease patients (95). These sterolomic data are compatible with the hypothesis that an absence of LIPA activity leads to reduced production of 25-HC, consequently removing a brake on mitochondrial-induced inflammasome activation with the ultimate result of an inflammatory phenotype. Viaud *et al.* (88) linked inflammasome activation with signaling through reactive oxygen species (ROS) (86, 88); this is supported by lalistat-treated macrophages showing increased mitochondrial ROS generation. Enhanced mitochondrial production of ROS could explain increased levels of cholestane-3β,5α,6β-triol in Wolman’s disease patients and would also fit the hypothesis of enhanced cholesterol biosynthesis in macrophages with insufficient LIPA activity. Importantly, we found levels of 5,6-epoxycholesterol, the immediate autoxidation product of cholesterol and precursor of cholestane-3β,5α,6β-triol, to be elevated in Wolman's disease plasma (95).

### Human patients with a deletion in both LIPA and CH25H

In 2017, Goenka *et al.* (96) reported the first case of patients with deletions of both *LIPA* and *CH25H*, two adjacent genes on chromosome 10. These patients showed susceptibility to Bacillus Calmette-Guérin (BCG) vaccine-associated abscesses. BCG is a vaccine primarily used against tuberculosis; it was developed from *Mycobacterium bovis*, and this bacterium was isolated by Goenka *et al.* (96) from infected lymph nodes (axillary lymphadenitis) of one of these double-deletion patients. In their report, Goenka *et al.* (96) described five patients with the double deletion, and four of five of these infants presented with localized BCG abscess. In contrast, no BCG abscesses were reported in eight children with *LIPA*-only mutation. We confirmed a complete absence of 25-HC in the plasma of the double-deletion patients. Significantly, there is no other report of a deficiency in CH25H in human. What might be the explanation for the susceptibility of patients with the double mutation toward BCG abscess? An abscess is defensive reaction against infectious material with the release of cytokines triggering an inflammatory response, so one explanation for these abscesses may be a consequence of a failure of 25-HC to repress inflammasome activation in macrophages. BCG is known to activate TLR4 and stimulate IFNγ production (97, 98), which will lead to macrophage biosynthesis of 25-HC in normal macrophages; however, an absence of CH25H enzyme will prevent this, resulting perhaps in overactivation of inflammasomes. Significantly, genetic deficiency in IFNγ or its receptor IFNGR (IFNγ receptor) enhances host susceptibility to *M. tuberculosis* (79, 99). This may be explained by a failure of macrophages to produce 25-HC and hence remove paracrine protection of epithelial cells from *M. tuberculosis* spread. This may be a second reason for BCG abscesses in the double-deletion infants, where a failure in 25-HC production could result in cell-to-cell spread of bacteria. Whatever the mechanism, the report by Goenka *et al.* (96) irrefutably links *CH25H* and 25-HC to resistance against bacterial infection in human.

## Antiviral activity of oxysterols

### IFN CH25H and 25-HC in the antiviral response

In 2011, soon after the seminal articles of Baumann *et al.* (3), Diczfalusy *et al.* (2), and Park and Scott (4) linking TLR, IFN, CH25H, and 25-HC to the immune response against bacterial infection (2, 3, 4), Blanc *et al.* (10) proposed a mechanism by which the host defends itself against viral infection through IFN-mediated downregulation of sterol biosynthesis. In genome-wide lipid-associated gene expression experiments on macrophages, they found negative regulation of the sterol pathway upon viral infection or treatment with IFNγ or IFNβ. nSREBP-2 was reduced upon viral infection or IFNβ treatment as were cholesterol levels after 48 h. Thus, in response to viral infection, IFN produced as part of the innate immune response downregulates the expression of genes of the cholesterol biosynthesis pathway, ultimately leading to reduced cholesterol synthesis (10). In a follow-on study, Blanc *et al.* (9) uncovered the link between IFN and reduced cholesterol biosynthesis to be 25-HC (Fig. 1). They showed that upon viral infection or IFN treatment of macrophages, stimulation of either IFNAR or IFNGR led to STAT1 activation, *Ch25h* transcription, and 25-HC synthesis and secretion. 25-HC was found to be a paracrine inhibitor of a broad range of viral infections, and the mechanism by which 25-HC limits viral infections was confirmed to involve in downregulation of the cholesterol biosynthesis pathway (9). Importantly, oxysterol profiling of mouse BMDM infected with mouse cytomegalovirus (mCMV), a double-stranded enveloped virus, or treated with IFNβ or IFNγ showed 25-HC to be the only hydroxycholesterol secreted into the medium or found in the cell pellet (9). Following IFN stimulation, the majority of 25-HC was secreted in the first 8 h of incubation, and *Ch25h* was found to be upregulated after 6 h. Neither *Cyp27a1* nor *Cyp7a1* was upregulated in response to IFNγ or mCMV explaining the absence of 26-HC and 7α-hydroxycholesterol (7α-HC) from stimulated macrophages. However, desmosterol and cholesterol levels in cells were found to fall with time indicating reduced flux through the cholesterol biosynthesis pathway in response to 25-HC. IC_50_ values for the antiviral role of 25-HC as measured by viral growth inhibition were found to vary between 20 nM (8 ng/ml) and 750 nM (300 ng/ml) depending on the virus and host cell. Enveloped viruses found to be inhibited include influenza A (H1N1), herpes simplex virus 1 (HSV-1), varicella-zoster virus, and murine gamma herpes virus 68. 25-HC was not found to inhibit adenovirus 5 or 19a, which are nonenveloped viruses (9). Interestingly, two other oxysterols that inhibit the processing of SREBP-2 and hence cholesterol biosynthesis, 26-HC and 24S,25-EC (34), also inhibit mCMV growth but at a higher IC_50_ than 25-HC (9). This observation highlights the importance of performing detailed sterolomic studies when studying oxysterol involvement in the immune response as multiple oxysterols may have additive effects on the response.

**Fig. 7:**
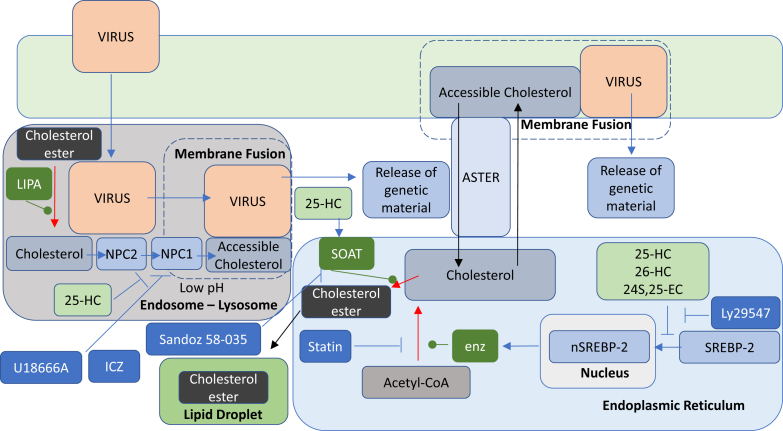
Membrane fusion and the antiviral activity of 25-HC. Symbols and colors are as in previous figures.

Beyond the effect of 25-HC on downregulating the expression of SREBP-2 target genes in infected cells, it is not clear how this translates into viral growth inhibition beyond reducing cellular cholesterol. Experiments with mCMV-infected mouse embryonic fibroblasts (National Institutes of Health [NIH]-3T3) treated with the potent synthetic LXR ligands GW3965 and T090317 indicated that LXR activation is not part of the antiviral response, at least against this virus and by this host cell. In contrast, the antiviral effect of 25-HC toward NIH-3T3 cells infected by mCMV was partially blocked by Ly295427 (9), a synthetic agent that blocks the suppression of SREBP processing mediated by oxysterols (Fig. 7) (100). The enantiomer of 25-HC was largely ineffective as a viral inhibitor (9), arguing that inhibition of viral growth is via interaction with proteins, for example, INSIG (Fig. 4) (34), rather than a direct effect of 25-HC interfering with membrane structure, that is, through its incorporation into the membrane. In the mCMV-NIH-3T3 context, the level of internalized mCMV genome was unaffected by 25-HC, arguing against viral entry as the primary mode of mCMV inhibition. Blanc *et al.* suggested that inhibition of early steps in sterol synthesis may be important for the antiviral effect of 25-HC, as in mCMV-infected mouse embryonic fibroblasts', viral growth inhibited by 25-HC (1 μM, 400 ng/ml) could be restored by addition by mevalonolactone, the lactone form of mevalonate, and by C_20_ geranylgeraniol, but not by C_15_ farnesol. This implicates protein prenylation by geranylgeraniol during viral infection with the antiviral effect mediated by 25-HC. However, at 5 μM 25-HC, the antiviral effect of 25-HC was maintained (9). A further important point uncovered in the studies of Blanc *et al.* was that the antiviral potency of 25-HC increased for most infections under lipid-depleted conditions, supporting the involvement of the cholesterol synthesis pathway (9). In lipid-replete media, cells can receive cholesterol by lipoprotein uptake reducing the requirement for new synthesis. Interestingly, the finding of Blanc *et al.* mirrors those of Potena *et al.* (101) who found that statins inhibit the replication of CMV in human endothelial cells, and that addition of mevalonate to treated cultures rescued statin-mediated inhibition of viral growth.

In summary, Blanc *et al.* (9) concluded that activation of pattern recognition receptors by the viral particle is sufficient to activate the transcription of the IFN-stimulated gene *Ch25h* via STAT-1 binding to the *Ch25h* promoter leading to 25-HC production and secretion and that it was likely that 25-HC mediates its antiviral effects through multiple levels of entry and growth depending on the host system and virus.

### IFN, CH25H, 25-HC, and membrane fusion

In an article published back to back with that of Blanc *et al.* (9), Liu *et al.* (8) proposed that 25-HC inhibits growth of enveloped viruses by blocking membrane fusion between the virus and host cell. Liu *et al.* confirmed that *Ch25h* is induced in mouse BMDM by TLR agonists, most potently by TLR3 and TLR4 agonists, and that both IFNα and IFNγ stimulate *Ch25h* expression (Fig. 1). When cells were preincubated with 25-HC at 1 μM (400 ng/ml), 25-HC was found to be antiviral against vesicular stomatitis Indiana virus (VSV), human immunodeficiency virus (HIV), Ebola virus, Nipah virus, Russian spring-summer encephalitis virus and Rift Valley fever virus, as well as HSV and murine gamma herpes virus 68 (8). In agreement with Blanc *et al.* (9), 25-HC was not found to inhibit growth of nonenveloped viruses (8). Liu *et al.* (8) reasoned that the antiviral effects of 25-HC were independent of LXR activation based on experiments in human embryonic kidney 293T (HEK293T) cells where the LXR ligand 22R-HC failed to have an antiviral effect against VSV. 22R-HC is also an inhibitor of SREBP-2 processing (34), so this finding also argues against the inhibition of the cholesterol biosynthesis pathway in the antiviral response, at least against VSV in HEK293T cells.

The studies by Liu *et al.* favored a hypothesis that 25-HC inhibits viral entry to the host cell by inhibiting fusion of the viral and host membranes, an essential step for an enveloped virus to replicate (Fig. 7). Using VSV as a model virus, they showed that 25-HC inhibited virus-cell fusion at 1 μM (400 ng/ml) in HEK293T cells. They found that the longer the preincubation time with medium derived from cells overexpressing *Ch25h* the greater the viral inhibition. Surprisingly, overexpression of SREBP-2 did not reverse the antiviral effect of 25-HC, nor did addition of mevalonate prior to 25-HC treatment (8). VSV undergoes pH-dependent viral entry, via obligatory endocytosis of the virus-receptor complex with exposure to acidic pH in the endosomal/lysosomal vesicle and fusion with the endosomal/lysosomal membrane, whereas HIV undergoes pH-independent entry (102). With pH-independent viral entry, the viral membrane directly fuses with the plasma membrane at neutral pH. Liu *et al.* found 25-HC to inhibit HIV entry to CEM cells, a lymphoblastic cell line, and concluded that 25-HC modifies cell membranes to impede viral infection. An important aspect of the study of Liu *et al.* was the demonstration in vivo of the antiviral activity of 25-HC. Humanized mice were given intraperitoneal injection of 25-HC (50 mg/kg) 12 h prior to infection with HIV, 25-HC was administered daily, and after 7 days, serum HIV RNA was reduced by 80% compared with control mice, which were given vehicle (8).

### 25-HC, 26-HC, IL-6, and the antiviral response

Although in this review we have so far focused on the anti-inflammatory action of oxysterols (84, 85, 88), certain oxysterols have also been assigned proinflammatory roles (7, 103, 104). Taking note of this, Cagno *et al.* (105) suggested that oxysterols may work as inducers of proinflammatory cytokines, which themselves may have an antiviral effect. To test this hypothesis, Cango *et al.* exploited HSV-1 infection as a model system in which an antiviral cytokine reaction is triggered in host cells by the virus itself. Cango *et al.* found that 25-HC and 26-HC had antiviral effects if added before or after viral infection. In fact, when added to Vero cells (African green monkey kidney cells) before infection, the half maximal effective concentration (EC_50_) determined for the antiviral effect of the two oxysterols was 5 μM (2 μg/ml) and 17 μM (6.8 μg/ml), respectively. However, when using a different assay, where 25-HC and 26-HC were added postinfection, the respective EC_50_ values were much lower at 180 nM (72 ng/ml) and 220 nM (88 ng/ml). Based on the knowledge that the proinflammatory cytokine IL-6 is produced in response to HSV-1 infection and evidence that it has an antiviral effect, Cango *et al.* tested if 25-HC or 26-HC pretreatment of cells would induce IL-6 secretion. Both oxysterols had this effect, as did viral treatment alone, and preincubation with either oxysterol followed by viral treatment proved to be additive toward IL-6 secretion in HeLa cells (105). Cango *et al.* then confirmed that IL-6 was antiviral against HSV-1 and proposed that one mechanism by which 25-HC and 26-HC may be protective against viral infection is through upregulation of antiviral IL-6 (Fig. 1). The explanation of the antiviral effect of these oxysterols postinfection was not obvious but could be due to sustained production of IL-6. Interestingly, earlier work from the same group had indicated that both 25-HC and 26-HC had antiviral activity against the nonenveloped viruses human papillomavirus-16, human rotavirus, and human rhinovirus, which argued against a general mechanism for antiviral activity of 25-HC involving membrane fusion, but rather these oxysterols may inhibit the viral replication machinery postviral entry (106).

### 25-HC and coronaviruses

IFNs are induced by coronaviruses (107), and IFN-stimulated genes expressed by cells infected with SARS-CoV and SARS-CoV-2 include *CH25H* (13, 14). SARS-CoV-2 infects lung epithelial cells, and *CH25H* has been shown to be upregulated in both epithelial cells and macrophages found in bronchioalveolar lavage fluid from COVID-19 patients (13). SARS-CoV-2 also infects intestinal epithelial cells (108), and Zang *et al.* (14) confirmed *CH25H* to be an IFN-stimulated gene in human enteroids. Importantly, 25-HC has been found to be elevated in some patients suffering from COVID-19 (12, 109). Zu *et al.* (12) performed a longitudinal study on a single COVID-19 patient analyzing serum samples for 25-HC by LC-MS (without hydrolysis) at four time points. 25-HC was stable at a level similar to controls (<10 ng/ml) for the first three time points but jumped to about 60 ng/ml, 2 days before the patient deceased. In a more detailed study of 144 adults with SARS-CoV-2, Marcello *et al.* (109) found that a group of patients with mild symptoms showed a modest but significant increase in serum 25-HC (9.7 ± 2.5 ng/ml, mean ± SD) compared with controls (8.5 ± 2.6 ng/ml), whereas the other patient groups displaying moderate (8.4 ± 2.6 ng/ml) or severe symptoms (7.6 ± 2.5 ng/ml) showed a small decrease in 25-HC levels. This study was performed by GC-MS for total 25-HC following hydrolysis of sterol esters. Interestingly, the serum concentration of 26-HC fell from 171.5 ± 34.6 ng/ml in controls to 142.3 ± 33.2 ng/ml in patients with mild symptoms to 119.6 ± 52.4 and 87.8 ± 31.9 ng/ml in patients with moderate and severe symptoms, respectively (109). Marcello *et al.* (109) suggested treatment with 26-HC as a possible course of action against severe COVID-19.

SARS-CoV-2 infection proceeds via the viral spike protein binding to the angiotensin-converting enzyme 2 (ACE2) receptor on the plasma membrane of host cells, followed by priming of the spike protein by the serine protease transmembrane protease/serine subfamily member 2 (TMPRSS2) prior to membrane fusion of the viral envelope with the plasma membrane of the host cell and release of viral RNA into the cell (110, 111). This is known as the early fusion pathway that dominates SARS-CoV-2 infection. An alternative pathway in cells not expressing membrane-bound TMPRSS2 is the endosomal pathway in which viral particles are endocytosed and the spike protein is primed for membrane fusion with the membrane of the lysosomal-endosomal compartment by a cathepsin cysteine protease, itself activated by the low pH in the lysosomal-endosomal compartment (Fig. 7) (110, 111).

Because of the high safety requirement of handling SARS-CoV-2, a replication-restricted pseudovirus, which bares the viral coat proteins, is often used in laboratory experiments of viral entry, and Wang *et al.* (13) exploited a SARS-CoV-2 pseudovirus in a study of viral entry into lung epithelial cells. SARS-CoV-2 entry to lung epithelial cells is primarily through the early fusion pathway rather than by endocytosis, and Wang *et al.* (13) found that pseudovirus entry to Calu-3 cells, an epithelial cell line expressing ACE2 and TMPRSS2, to be inhibited by 25-HC at an IC_50_ of 550 nM (220 ng/ml). They then showed that 25-HC acts at the level of spike-mediated membrane fusion rather than receptor binding, and by exploiting ALOD4 showed that 25-HC blocks viral entry to Calu-3 cells by mobilizing accessible cholesterol away from the plasma membrane. Significantly, supplementation of cholesterol using a cyclodextrin vehicle restored SARS-CoV-2 fusion and entry (13). Similar effects were observed with SARS-CoV and Middle East respiratory syndrome-related CoV pseudoviruses (13).

As discussed previously, multiple mechanisms exist by which 25-HC can alter the level of plasma membrane accessible cholesterol. Wang *et al.* (13) favored a mechanism by which 25-HC activated SOAT, by removing nonesterified cholesterol from the endoplasmic reticulum and encouraging net transport of accessible cholesterol from the plasma membrane to the endoplasmic reticulum. They found that the SOAT inhibitor, Sandoz 58-035, increased the amount of accessible cholesterol in the plasma membrane of 25-HC-conditioned Calu-3 cells, and importantly rescued SARS-CoV-2 pseudovirus entry, as did SOAT knockdown by shRNA (13). These experiments were performed in lipid-depleted medium; hence, cholesterol uptake via the LDL receptor was restricted, so its regulation by SREBP-2 was not considered. In summary, the results of Wang *et al.* (13) indicate that the early membrane fusion pathway involves plasma membrane accessible cholesterol, and its depletion by 25-HC-mediated activation of SOAT restricts viral entry and viral genome release (Fig. 7).

Zang *et al.* (14) also utilized a SARS-CoV-2 pseudovirus to demonstrate the antiviral properties of 25-HC. They found that 25-HC inhibited pseudovirus infection of MA104 cells, a Rhesus monkey epithelial cell line, at an EC_50_ of 1.5 μM (600 ng/ml) and that WT SARS-CoV-2 was also inhibited by 25-HC in an assay of HEK293-hACE2 cells (HEK293 cells expressing the human ACE2 receptor) (14). To study the mechanism of 25-HC antiviral activity against SARS-CoV-2, they exploited an in vitro cell fusion assay and found that both *CH25H* expression and addition of 25-HC reduced membrane fusion. Fluorescent-labeled 25-HC (C4 TopFluor-25-HC) was found to have almost identical antiviral activity against the SARS-CoV-2 pseudovirus in HEK293 cells as 25-HC and similarly blocked membrane fusion. Interestingly, C4 TopFluor-25-HC was found to accumulate in the late endosome-lysosome compartment when added to HEK cells in medium containing fetal bovine serum. 25-HC treatment of these cells led to intracellular accumulation of TopFluor-cholesterol and of nonesterified cholesterol as measured by filipin staining, a similar effect to that mediated by the NPC1 inhibitors U18666A and itraconazole (ICZ). Importantly, ICZ significantly reduced SARS-CoV-2 pseudovirus titers, and the antiviral activities of 25-HC and ICZ were found to be diminished in serum-free media. In combination, these data suggested to Zang *et al.* that 25-HC inhibits the endosomal pathway of viral infection and that nonesterified cholesterol accumulates in the endosomal/lysosomal compartment as a consequence of NPC1 inhibition. Cholesterol accumulation in the interior of the compartment was reasoned to mediate the antiviral activity of both 25-HC and ICZ (14). A complication to this mechanism is that in cellular assays, 25-HC travels directly to the endoplasmic reticulum without traversing the lysosome on its way to inhibiting SREBP-2 processing (34, 112); however, in vivo, it is likely that when derived from the circulation, where the majority of 25-HC is esterified (113, 114), the ester as part of lipoprotein particles is taken up by receptor-mediated endocytosis and travels to the lysosome where it is hydrolyzed to the nonesterified molecule. Zang *et al.* (14) concluded that 25-HC reduces SARS-CoV-2 spike-mediated fusion via a mechanism that involves altered cholesterol levels leading to inhibition of viral replication. The data by Zang *et al.* (14) suggest that inhibition of NPC1 by 25-HC leads to a reduction in accessible cholesterol in the membrane of the endosomal/lysosomal compartment, which results in reduced membrane fusion with the viral envelope and thereby preventing release of viral RNA.

In summary, the studies of Wang *et al.* (13) and Zang *et al.* (14) suggest a model in which viral infection leads to activation of pattern recognition receptors on macrophages, IFN secretion, upregulation of *CH25H*, and synthesis and secretion of 25-HC. Paracrine or autocrine, or even hormonal signaling, by 25-HC leads to reduced accessible cholesterol in the plasma membrane and/or endosomal/lysosomal membrane of host cells restricting SARS-CoV-2 spike protein-mediated fusion and viral replication.

Following on from the work of Zu *et al.* (12), Wang *et al.* (13), Zang *et al.* (14), and Marcello *et al.* (109) connecting 25-HC with the mechanism of SARS-CoV-2 infection, others have suggested therapeutics based on the 25-HC structure (115, 116). Kim *et al.* (115) prepared nanovesicles made of 25-HC and didodecyldimethylammonium bromide (DDAB), that is, 25-HC@DDAB, DDAB being a cationic lipid that forms liposome-like structures. 25-HC@DDAB was found to selectively accumulate in lung tissue and downregulate the NF-κB and SREBP-2 pathways and in peripheral blood mononuclear cells derived from COVID-19 patients, reducing inflammatory cytokine levels (115). Ohashi *et al.* (116) screened a panel of naturally occurring and semisynthetic oxysterols for anti-SARS-CoV-2 activity using a cell culture infection assay. They found that 7-oxocholesterol (7-OC, also called 7-ketocholesterol), 22R-HC, 24S-HC, and 26-HC inhibited SARS-CoV-2 propagation in cultured cells. Two semisynthetic oxysterols based on the 20-HC skeleton, but *i*) with a C_4_ side chain (rather than the C_8_ as in cholesterol) attached at the terminal carbon to a pyridine ring through the carbon at position 3 of the aromatic heterocycle (Oxy210, see supplemental data) and *ii*) with a 5α-reduced B-ring and a C_5_ side chain with a C-21 ethyl rather than methyl group (Oxy232), were found to display more robust anti-SARS-CoV-2 activities, reducing viral replication more than 90% at 10 μM and 99% at 15 μM, respectively (116). These studies suggest a therapeutic value for side-chain oxysterols and their derivatives in the treatment of SARS-CoV-2.

With respect to 7-OC, Ghzaiel *et al.* (117) proposed that 7-OC may be involved in the pathophysiology of SARS-CoV-2 by promoting acute respiratory distress syndrome, and therapies countering the toxic effects of 7-OC may have therapeutic merit.

## Proinflammatory oxysterols

As mentioned previously, 25-HC can have a proinflammatory as well as an anti-inflammatory role. It can augment macrophage and epithelial cell secretion of the inflammatory cytokines IL-6, IL-8, and macrophage colony-stimulating factor in a mechanism involving stimulation of TLR, but which is otherwise ill defined (7, 104, 118). As mentioned previously, 26-HC may also augment inflammatory cytokine production (105), one mechanism may involve its action as a selective estrogen receptor modulator (119). The concentration of available oxysterols is likely to dictate whether they behave in a proinflammatory manner or an anti-inflammatory manner, and it is possible that multiple oxysterols have additive effects. It is noteworthy that 25-HC repressed the expression of *Il1b* in LPS-stimulated *Ch25h*^−/−^ macrophages at a level of 100 nM (40 ng/ml) (84), whereas some proinflammatory effects of 25-HC are observed at micromolar concentrations (104, 118).

Besides side-chain oxysterols, ring oxysterols also have modulatory effects on the immune system. 7β-Hydroxycholesterol (7β-HC) has been found to enhance IL-8 mRNA in, and secretion from, U937 human promonocytic leukemia cells (120), whereas 4β-HC has been implicated in colon inflammation (121). However, it is oxysterols modified in both the ring and side chain that have attracted most attention in immunology and which are discussed later.

## 7α,25-diHC and EBI2 (GPR183)

EBI2 is a G protein-coupled receptor (GPR183); it is widely expressed in the immune system by multiple cell types, including macrophages, neutrophiles, B cells, T cells, and DCs (7, 122). The ligands for GPR183 were discovered independently by scientists from Novartis and Johnson & Johnson to be dihydroxycholesterols with the most potent being 7α,25-diHC and 7α,26-dihydroxycholesterol (7α,26-diHC), presumably the 25R-epimer, although it is not clear whether the 25S-epimer also has biological activity (5, 6). The reader can, however, assume 25R stereochemistry unless stated otherwise, as this is normally the dominant epimer in human and mouse. While the ligand identifications made by Hannedouche *et al.* (5) were made based on chromatographic separations and detailed MS analysis of biologically active fractions from sheep liver, the identifications made by Liu *et al.* (6) were perhaps fortuitous as GC-MS identification from porcine spleen were of 7α-HC and 7β-HC, the true ligands only being identified following screening of a panel of synthetic oxysterols. Liu *et al.* (6) went on to measure by GC-MS the level of 7α,25-diHC to be 1.4 ± 0.2 ng/g of spleen tissue, which increased to 9.4 ± 1.3 ng/g after LPS challenge and determined a dissociation constant for 7α,25-HC from GPR183 to be 450 pM (180 pg/ml). For comparison, 100 μg of 7α,25-diHC was isolated by Hannedouche *et al.* (5) from 35 kg of pig liver; this translates to a 7α,25-diHC concentration of 2.9 ng/g of tissue. Both groups found 7α,25-diHC and 7α,26-diHC to be chemoattractants to cells expressing GPR183, with 7α,25-diHC as the more potent ligand (5, 6).

### B cells

**Fig. 8:**
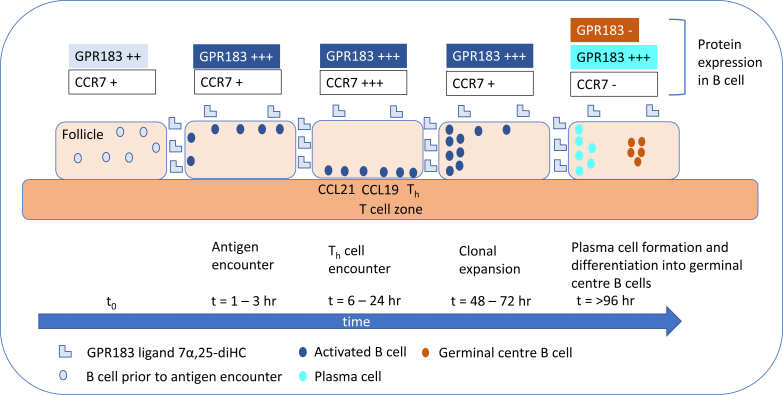
GPR183 and 7α,25-diHC guide B-cell positioning within lymphoid tissue. Figure inspired by Refs. (5, 6, 7). On a time course from antigen encounter at time 0 to 4 days post exposure, the guidance of activated B cells towards 7α,25-diHC and chemokines CCL19 and CCL21 according to expression of their respective receptors GPR183 and CCR7 is displayed.

An important function of 7α,25-diHC, 7α,26-diHC, and GPR183 is in the guidance of immune cells required for mounting an adaptive immune response (5, 6, 7). The first described biological activity of GPR183-expressing cells toward these oxysterols was of B cells responding to antigen encounter (5, 6, 7). This is eloquently described by Cyster *et al.* (7) and will be precised here. In the spleen, following antigen encounter and receptor engagement, B cells upregulate expression of GPR183. Activated B cells then migrate toward 7α,25-diHC, which is suggested to be most abundant, based on enzyme expression data, in the inter and outer follicular regions of lymph tissue (Fig. 8). Within a few hours of antigen encounter, GPR183 promotes B-cell positioning toward the outer region of the follicle, close to where antigens enter the follicle. After about 6 h, activated B cells are distributed along the interface between the B-cell follicle and the T-cell zone in the spleen in response to the chemokine ligands CCL19 and CCL21 in the T-cell zone and upregulation of their receptor CCR7 in the B cells. The interface between the B-cell follicle and T-cell zone is a region in which B cells encounter cognate T_h_ cells. Signals from T_h_ cells maintain GPR183 expression but downregulate the chemokine receptor CCR7 for CCL19 and CCL21. Next after 2–3 days, B cells are positioned in a GPR183-dependent manner toward the outer regions of the follicle, with a preference for interfollicular regions. It is suggested that 7α,25-HC is most abundant in these regions. The reason for this B-cell positioning may be to increase exposure to newly arriving antigen and/or for DCs in these areas to augment plasmablast responses and plasma cell formation. During differentiation into germinal center B cells, GPR183 is downregulated important for movement to the follicle center. In the germinal center, B cells undergo rounds of proliferation. Importantly, deficiency in GPR183 leads to a reduced plasma cell response and antibody secretion (7). GPR183 deficiency in mice leads to reduced immunoglobulin G and immunoglobulin M antibody response (123, 124).

The distribution of naive B cells within the follicle is also influenced by GPR183, favoring migration to the outer follicle edge. Besides B cells, CD4^+^ T cells also express GPR183 and migrate toward 7α,25-diHC (6).

### DCs

**Fig. 9:**
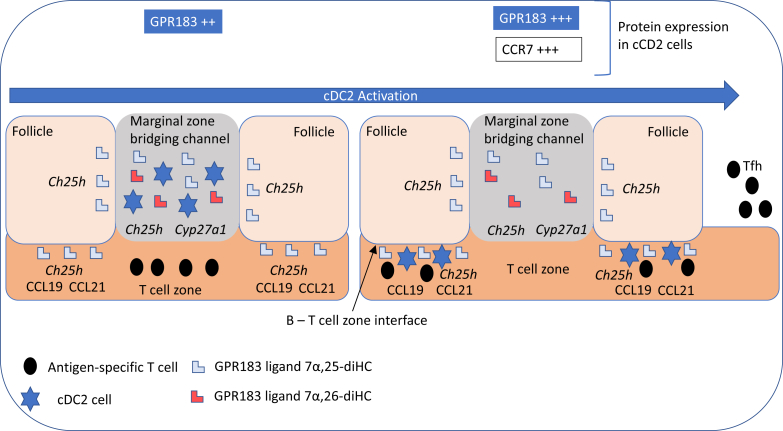
Positioning of naive and activated cDC2 cells to promote T follicular helper (Tfh) cell differentiation. Naive cDC2 cells are positioned in marginal zone bridging channels by the GPR183 ligands 7α,25-diHC and 7α,26-diHC. Upon activation, CCR7 is expressed, and cDC2 cells are guided by 7α,25-diHC to the B-T cell zone interface where they interact with early activated T cells to promote Tfh cell differentiation. Tfh cells help B cells produce antibodies against foreign pathogens. Figure inspired by Ref. (127).

DCs, the antigen-presenting cells of the innate and adaptive immune systems, express GPR183 and in vitro migrate toward 7α,25-diHC (5, 6, 125, 126). In 2013, articles by Gatto *et al.* (125) and Yi and Cyster (126) established that DCs in the spleen require GPR183 and 7α,25-diHC for proper positioning in marginal zone bridging channels, a specialized type of interfollicular region, and are important for mounting T cell-dependent antibody responses against some blood-borne antigens (7). In the spleen, there are two types of DC cells, cDC2 cells important for presenting antigen to CD4^+^ T cells and cDC1 cells that present antigen to CD8^+^ T cells. Naive cDC2 cells are mostly located in the marginal zone bridging channels (Fig. 9). Recently, Lu *et al.* (127) provided evidence that not only 7α,25-diHC but also 7α,26-diHC is important in positioning of cDC2 cells. *Cyp27a1* is expressed in the marginal zone bridging channels along with *Ch25h*, and Lu *et al.* (127) proposed that under stable or homeostatic conditions, 7α,25-diHC and 7α,26-diHC in combination attract cDC2 cells to these channels. After cDC2 cell activation, GPR183 and CCR7, the receptor for the T-cell zone chemokines, CCL19 and CCL21, are upregulated directing activated cDC2 cells toward in the T-cell zone where 7α,25-diHC helps position the cells at the B-T zone interface, where they can interact with early activated T cells and promote differentiation to T follicular helper cells (127). T follicular helper cells help B cells produce antibodies against foreign pathogens and are found in the B-cell zone of secondary lymphoid organs. Lu *et al.* (127) measured the 7α,26-diHC content of spleen extracts by LC-MS and found it to be present at a level of 12 ng/g (30 nM) in WT mice, whereas 7α,26-diHC was absent in extracts from *Cyp27a1*^−/−^ mice. In confirmation of the importance of *Cyp27a1* in DC positioning, *Cyp27a1/Ch25h* double knockout mice showed a more severe cDC2 deficiency than *Ch25h*^−/−^ mice and more comparable to *Gpr183*^−/−^ mice. cDC1 cells may also be important for the proper positioning of activated cDC2 cells toward the outer T-cell zone by acting as a sink for 7α,25-diHC (and 7α,26-diHC) more centrally within the T-cell zone through conversion of the dihydroxycholesterols to their 3-oxo-4-ene derivatives and inducing a gradient of 7α,25-diHC maximizing at the outer T-cell zone. Interestingly, type I IFNs can negatively regulate DC positioning in the outer T-cell zone by upregulating *Ch25h* expression and presumably disturbing 7α,25-diHC gradients in the T-cell zone (127).

In summary, GPR183-mediated positioning of B and DC cells and possibly CD4+ T cells in the interfollicular region may promote interactions between cells that together augment the antibody response (5, 6, 7).

### 7α,25-diHC, 7α,26-diHC, enzymes, and genes

While Hannedouche *et al.* (5) and Liu *et al.* (6) made MS-based measurements of 7α,25-diHC from liver (pig liver 3 ng/g) and lymphoid tissue (mouse spleen 1–10 ng/g) (5, 6), there are few other quantitative measurements in the literature of this or of the other major GPR183 ligand 7α,26-diHC in tissue or fluids (121, 127, 128, 129, 130). Lu *et al.* (127) measured 7α,26-diHC by LC-MS to be at a level of 12 ng/g in mouse spleen tissue, whereas Wanke *et al.* (128) found 7α,25-diHC to be elevated in central nervous system (CNS) tissue from an EAE mouse model but did not provide concentration units; similarly, Mutemberezi *et al.* (129) measured 7α,25-diHC in the context of neuroinflammation but failed to provide concentration units. Guillemot-Legris *et al.* (121) performed extensive oxysterol profiling by LC-MS of colon and liver tissue in the context of a mouse model of colitis and found 7α,25-diHC to be at a level of 100–200 pmol/g tissue (40–80 ng/g) in colon and about 20 pmol/g (8 ng/g) in liver. Raselli *et al.* (131) measured oxysterol levels in human and mouse livers by LC-MS but failed to detect 7α,25-diHC. The level of 7α,26-diHC in human liver was about 3 ng/g, but in liver from patients with nonalcoholic steatohepatitis, the downstream metabolite 7α,(25R)26-dihydroxycholest-4-en-3-one (7α,26-diHCO) was significantly elevated above control values, that is, 21 ng/g cf. 4 ng/g. Interestingly, these results were not replicated in a mouse model of nonalcoholic steatohepatitis, where levels of 7α,26-diHC and 7α,26-diHCO were about 25 and 50 ng/g, respectively, in both WT and treated animals (131). It is important to note that both 7α,26-diHC and 7α,26-diHCO are found in man and mouse as 25R-epimer and 25S-epimer where the stereochemistry at C-25 is different (113, 132), and that Raselli *et al.* (131) specifically quantified the 25R-epimer. Very few other studies define the specific epimer(s) measured; however, the 25R-epimers is usually dominant except in the case of CYP27A1 deficiency (113, 132, 133), where the 25S-epimer dominates. By default, and unless explicitly stated here, it can be assumed that the stereochemistry at C-25 is R, and the concentrations presented are for the 25R-epimer or for a combination of C-25R and C-25S epimers. This is also true with respect to measurements of biological activity where few if any reports define the stereochemistry at C-25, and the reader is left to assume 25R stereochemistry. Even the patent filed by Novartis of the invention of 7α,26-diHC as a ligand of EBI2 (GPR183) failed to define the stereochemistry at C-25 (WO 2010/066689 A2). To the best of our knowledge, the biological activity of 7α,(25S)26-diHC has not been tested toward GPR183.

Both 7α,25-diHC (1 ng/ml) and 7α,26-diHC (6–10 ng/ml) can be detected by LC-MS in human plasma following ester hydrolysis as the sum of esterified and nonesterified sterols (113, 134), but their levels as free nonesterified molecules are very low (7α,25-diHC, 0.2 ng/ml; 7α,26-diHC, 0.5 ng/ml (135)). However, the nonesterified 3-oxo-4-ene metabolites (produced through the action of HSD3B7) are more abundant (7α,25-diHCO, 1–2 ng/ml; 7α,26-diHCO, 3–8 ng/ml (113, 135)). In mouse plasma, the situation is similar with levels of nonesterified 7α,25-diHC and 7α,26-diHC being very low at about 0.1 ng/ml, with 7α,25-diHCO and 7α,26-diHCO at about 1 ng/ml (132). Nonesterified 7α,25-diHC and 7α,26-diHC are hardly detectable by LC-MS in “control” human cerebrospinal fluid (CSF) (<0.01 ng/ml), although their levels are measurable at about 0.05 ng/ml in CSF from people with pathogen-based infections of the CNS (135). The HSD3B7-derived metabolites 7α,25-diHCO and 7α,26-diHCO are detectable in control CSF at about 0.03 ng/ml, and from patients with pathogen-based infections, 7α,26-diHCO is elevated significantly to 0.05 ng/ml (135). In hydrolyzed “control” CSF, the sum of esterified and nonesterified 7α,25-diHC is about 0.05 ng/ml, whereas that of 7α,(25R/S)26-diHC is about 0.2 ng/ml (113). Note that concentrations of 7,25-diHCO and 7,26-diHCO are difficult to measure by LC-MS in hydrolyzed samples as the 7-hydroxy-3-oxo-4-ene structure dehydrates under the basic conditions of hydrolysis. In mouse CSF, unesterified 7α,25-diHC and 7α,26-diHC have only been measured by LC-MS in combination with their 3-oxo-4-ene analogs, concentration being in the region of 0.01–0.04 ng/ml (136).

The deficit in measurement of GPR183 ligand concentrations, particularly in tissue samples, has meant that gradients of 7α,25-diHC and 7α,26-diHC have been assumed based on expression of genes or enzymes required for their synthesis from cholesterol. These assumptions have been based on the hypothesis that CH25H and CYP7B1 are the dominant enzymes for 7α,25-diHC formation (Fig. 2A) and CYP27A1 and CYP7B1 for the formation of 7α,26-diHC (Fig. 2B). While this may be true in spleen (126), this is not necessarily correct in other lymphoid tissues as 7α,26-diHC can be synthesized from cholesterol via CYP7A1 to generate 7α-HC hepatically and then by CYP27A1 to generate 7α,26-diHC in the periphery (Fig. 2B), and it should be noted that 7α-HC is abundant in the circulation as the nonesterified molecule (5–20 ng/ml) (113, 114, 135) and can cross plasma membranes (52). Similarly, 7α,25-diHC can be generated from 7α-HC, the 25-hydroxylase being by CYP3A4 (132).

With respect to positioning of B cells in lymphoid tissue, Yi *et al.* (137) found that lymphoid stromal cells were the main CH25H-expressing and CYP7B1-expressing cells required for B-cell positioning, and that CH25H and CYP7B1 were abundant in interfollicular and outer follicular stromal cells but low in the central region of the follicle, thereby explaining movement of GPR183-expressing B cells to the outer and interfollicular regions when mounting the adaptive immune response (Fig. 8). CH25H, CYP7B1, and HSD3B7 were also found to be expressed in the T-cell zone of lymphoid tissue providing a route to 7α,25-diHC formation, then removal, and to the generation of 7α,25-diHC gradients (126).

Both *Ch25h*^−/−^ and *Cyp7b1*^−/−^ mice phenocopy *Gpr183*^−/−^ mice with respect to B-cell positioning and to a reduced T cell-dependent antibody response (5, 137) and also show a reduction in CD4^+^ DC numbers (125, 137). Interestingly, *Hsd3b7*^−/−^ mice have similar defects in B and DC cells as *Gpr183*^−/−^ mice emphasizing that it is molecular gradients that are the key to the T cell-dependent antibody response (137).

In human, CYP7B1 deficiency can present as oxysterol 7α-hydroxylase deficiency in the infant and spastic paraplegia type 5 (SPG5) in the adult (138). Oxysterol 7α-hydroxylase deficiency presents with liver disease, which can be successfully treated by bile acid replacement therapy (139), whereas SPG5 is a disorder with progressive degeneration of upper motor neurones leading to lower extremity weakness and spasticity. Neither SPG5 nor oxysterol 7α-hydroxylase deficiency has been explicitly associated with a defective antibody response; however, the first infant diagnosed with oxysterol 7α-hydroxylase deficiency died after liver transplant of Epstein-Barr virus-associated disseminated lymphoproliferative disease in all body tissues (140). It is perhaps noteworthy that lymphoproliferative disease is a B-lymphocyte growth and that GPR183 was first identified in a screen of upregulated genes in human B cells infected with Epstein-Barr virus (141).

Cerebrotendinous xanthomatotisis (CTX) is the human disorder resulting from CYP27A1 deficiency (133, 138). In infants, it can present with cholestatic liver disease and in later life as a neurological disease. There appear to be no specific associations between CTX and a compromised antibody response. HSD3B7 deficiency presents with cholestasis, but like CTX, it can be treated successfully by bile acid replacement therapy (138). The absence of a defective antibody response in people with deficiency in CYP7B1, CYP27A1, or HSD3B7 presents a conundrum, and once again, a major difference between human and mouse is evident. Significantly, in this context, neither *Cyp27a1*^−/−^ nor *Cyp7b1*^−/−^ mice phenocopy the human enzyme deficiencies other than in oxysterol patterns.

### Biology of GPR183 and 7α,25-diHC

There is good evidence that GPR183 and 7α,25-diHC are linked to inflammation of the colon (121, 130). In a study of colitis in human and mouse models of the disease, Wyss *et al.* (130) established that genes coding for GPR183, CH25H, and CYP7B1 were upregulated in colon tissue, with CYP27A1 also being upregulated in inflamed tissue from patients with ulcerative colitis. Concentrations of 7α,25-diHC and 7α,26-diHC were determined by LC-MS to be elevated in tissue from mouse colon and liver in an acute disease model, although it was unclear what units of concentration were exactly reported (130). It is interesting to note that Wyss *et al.* (130) found that 25-HC, 7α,25-diHC, and 7β,25-dihydroxycholesterol (7β,25-diHC) were present in *Ch25h*^−/−^ mouse tissue, explained by the sterol 25-hydroxylase activity of other enzymes besides CH25H (Fig. 2A). Wyss *et al.* (130) also established that *Gpr183*^−/−^ mice had fewer lymphoid structures than control animals, and defects in inflammation induced lymphoid structures, leading to the conclusion that the GPR183-oxysterol axis promotes the development of lymphoid structures and colitis. This agreed with work by Emgård *et al.* (142) who showed that expression of *Gpr183* and oxysterol-synthesizing genes *Ch25h* and *Cyp7b1* promoted the formation of colonic lymphoid tissues in the steady state and during inflammation. In an independent study of colitis, Guillemot-Legris *et al.* (121) found both 25-HC and 7α,25-diHC to be elevated in colon from mice models of colitis as was 4β-HC. In chronic mouse models of colitis, levels of 7α,25-diHC and 4β-HC were at a level of about 200 pmol/g (80 ng/g), about double that found in control animals.

Multiple sclerosis is an autoimmune disease where circulating autoreactive T cells escape immune regulation and enter the brain resulting in inflammation and demyelination. Interestingly, Chalmin *et al.* (143) found the migration of GPR183-expressing memory CD4^+^ T cells toward 7α,25-diHC to be linked with EAE, the mouse model of multiple sclerosis. In the context of the human biology, Clottu *et al.* (144) have shown that the functional GPR183 protein is expressed on human lymphocytes and that memory CD4^+^ T and B cells express more GPR183 than their naive counterparts (which unlike memory cells have not encountered antigen) and that in transwell assays memory CD4^+^ T cells are most responsive toward 7α,25-diHC. Interestingly, Clottu *et al.* (144) found a positive correlation between GPR183 expression and migration toward 7α,25-diHC in relapsing remitting multiple sclerosis in CD4^+^ and CD8^+^ memory T cells, a correlation not observed in the corresponding cells from healthy donors. This led to the suggestion that a difference in migration could indicate a difference in GPR183 expression during autoimmunity. However, Crick *et al.* (135) using LC-MS failed to find an elevation in the 7α,25-diHC or 7α,26-diHC in CSF or plasma of patients suffering from either relapsing remitting multiple sclerosis or clinically isolated syndrome. They did, however, find a reduction in the plasma content of 25-HC, perhaps disturbing the production of 7α,25-diHC in lymphoid tissue and disorientating homeostatic concentration gradients (135).

In the context of multiple sclerosis and EAE, Wanke *et al.* (128) found that GPR183 is highly expressed in inflamed white matter lesions in human multiple sclerosis brain, whereas in EAE mouse models, GPR183 promotes early migration of inflammatory CD4^+^ T cells into the CNS. They found that the majority of naive CD4^+^ T cells express GPR183, and that inflammatory Th17 (or T_h_17) cells derived from naive CD4^+^ T under IL-1β-induced and IL-23-induced differentiation maintain GPR183 expression. Their results suggest a role for GPR183 in early migration of encephalitogenic Th17 cells from peripheral lymphoid organs to the CNS. In the EAE mouse model, *Ch25h* and *Cyp7b1* were found to be upregulated in spinal cord, whereas *Hsd3b7* to be downregulated; this led to an increase in the GPR183 ligand and chemoattractant 7α,25-diHC in EAE animals (128). Using LC-MS, it was found that besides 7α,25-diHC, 7α,26-diHC and 7α,24-dihydroxycholesterol were also elevated in spinal cord from EAE animals, as was 25-HC, but levels of 24-HC and 26-HC were reduced (128). Interestingly, during EAE, microglia the macrophages of the brain, strongly express *Ch25h*, whereas *Cyp7b1* is expressed by infiltrating lymphocytes and monocytes but not microglia. *Hsd3b7* was found to be expressed in both groups of cells. Importantly, human Th17 cells express GPR183, and CD4^+^ T cells migrate toward 7α,25-diHC in a dose-dependent manner. In summary, the work of Wanke *et al.* (128) supports the hypothesis that in multiple sclerosis GPR183 and 7α,25-diHC promote the redistribution of inflammatory Th17 cells from secondary lymphoid organs to sites of inflammation.

It should also be noted that besides B cells, T cells, and DCs, GPR183 is also expressed in human astrocytes and promotes their in vitro migration (145) and also in primary human macrophages (146).

## RORγ1 and RORγ2 nuclear receptors

There are three retinoic acid-related orphan receptors (RORs): RORα (NR1F1), RORβ (NR1F2), and RORγ (NR1F3). RORγ has two isoforms, RORγ1 and RORγ2, the first isoform is simply called RORγ, whereas the second one is often called RORγt. RORγt has a shorter N terminus than RORγ, but otherwise, the domains are identical. RORγt is highly expressed in thymus and is an essential transcription factor for the development of proinflammatory IL-17-expressing Th17 cells. As discussed previously, Th17 cells are implicated in multiple sclerosis and its mouse model EAE. Although RORγt is expressed in multiple organs, they do not include the brain, but RORγt immunoreactive cells have been found in the meninges of multiple sclerosis patients, perhaps by infiltrating immune cells (147).

**Fig. 10:**
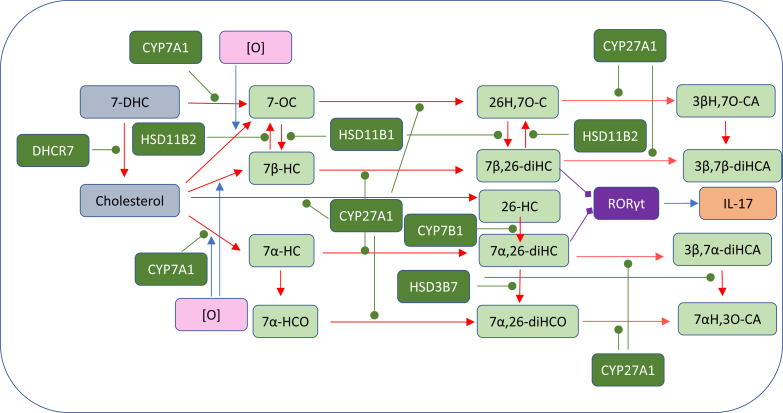
Pathway map detailing the biosynthesis and metabolism of the RORγ agonists 7α,26-diHC and 7β,26-diHC. Nonenzymatic oxidation is indicated by [O] on a pink background. 7α-HC, 7β-HC, and 7-OC have been found to be inverse agonists of RORγ.

Although LXRs are generally considered as the oxysterol nuclear receptors, oxysterols can also bind to the ligand binding domain (LBD) of RORγ and RORγt. In 2010, Wang *et al.* (148) found 7α-HC, 7β-HC, and 7-OC to be inverse agonists of RORγt (Fig. 3B). These three oxysterols can all be formed by autoxidation (92, 93), which may be not only ex vivo but also in vivo, as evidenced by their presence, and that of their downstream metabolites, in plasma from patients suffering from the cholesterol transport disorder NPC1 disease and also other lysosomal storage disorders (95). These three oxysterols can also be formed enzymatically: 7α-HC by CYP7A1 catalyzed oxidation of cholesterol (149); 7-OC by CYP7A1-mediated oxidation of 7-DHC (150, 151); and 7β-HC by reduction of 7-OC by HSD11B1 (Fig. 10) (152, 153, 154). The latter two oxysterols can be generated enzymatically in Smith-Lemli-Opitz syndrome, where 7-DHC is abundant on account of a deficiency in DHCR7 (155). Wang *et al.* (148) also found 24S-HC and 24S,25-EC to be inverse agonists of RORγ, but Jin *et al.* (156) also in 2010 reported 20S-HC, 22R-HC, and 25-HC to be agonists toward RORγ (Fig. 3B).

While 7α,26-diHC and 7β,26-dihydroxycholesterol (7β,26-diHC) are both ligands toward GPR183 (5), they have also been shown to be agonists toward RORyt (Fig. 3B) (157). Interestingly, while 7α,26-diHC is the more potent ligand toward GPR183, the reverse is true with respect to activation of RORγt, where 7β,26-diHC is more potent. Soroosh *et al.* (157) found that both ligands, presumably the 25R-epimers, reversed the inhibitory effects of ursolic acid in a cell-based reporter assay and in primary cells enhanced the differentiation of IL-17-producing cells in an RORγt-dependent manner. They found that in vivo administration of 7β,26-diHC to mice enhanced IL-17 production and that differentiating Th17 but not Th1 cells generate these two oxysterols (157). Soroosh *et al.* found *Cyp27a1* to be enriched in mouse Th17 cells, and when treated with [^3^H]7β-HC or [^3^H]7α-HC, these cells produced [^3^H]7β,26-diHC and [^3^H]7α,26-diHC, respectively, the reaction product [^3^H]7β,26-diHC being more readily formed (Fig. 10). Both 7β,26-diHC and 7α,26-diHC were shown using LC-MS to be endogenous molecules in mouse spleen, the level of the former isomer to be about 3% of the latter. 26-Hydroxy-7-oxocholesterol (26H,7O-C, also called 7-keto-27-hydroxycholesterol) was not detected in mouse spleen (157) but is a difficult molecule to detect by LC-MS (158); however, it also reverses the inhibitory effects of ursolic acid in a cell-based RORγt LBD reporter assay, but unlike 7α,26-diHC and 7β,26-diHC, 26H,7O-C was found to be largely inactive in full-length RORγ or RORγt reporter assays. Although not targeted by LC-MS analysis of mouse spleen, the CYP27A1 downstream metabolites of 7α,26-diHC and 7β,26-diHC, that is, the cholestenoic acids 3β,7α-diHCA and 3β,7β-diHCA (Fig. 10) were found to display minimal agonist activity in the LBD reporter assay; however, in this assay, the HSD3B7-derived metabolite 7α,26-diHCO showed similar activity to 7α,26-diHC (157). In support of the lack of activity of 26H,7O-C in the full-length RORγt reporter assay, 26H,7O-C was much less efficacious than 7β,26-diHC or 7α,26-diHC in promoting IL-17 producing mouse or human Th17 cells (157). These data suggest that conversion of dihydroxycholesterols to their 7-one equivalents may be a route to inactivation of RORγt ligands, as may further CYP27A1-mediated metabolism to cholestenoic acids.

Soroosh *et al.* (157) suggested that 7α,26-diHC, 7β,26-diHC, and 26H,7O-C were derived from 26-HC, the former by CYP7B1-mediated 7α-hydroxylation and the latter two via somewhat unlikely autoxidation reactions. An alternative route to the formation of 7α,26-diHC is via CYP27A1-mediated oxidation of 7α-HC, which is prevalent in the circulation, whereas the latter two oxysterols can be formed by CYP27A1-mediated oxidation of 7β-HC and 7-OC (Fig. 10) (95, 155).

A study by Beck *et al.* (159) in 2019 shed further light on the metabolism of 7β,26-diHC in the context of RORγt signaling. Following on from earlier work, which showed that 7β-HC and 7-OC are interconvertible by HSD11B enzymes, the 11B1 enzyme driving reduction and the 11B2 enzyme oxidation (152, 153, 154), Beck *et al.* (159) found that 7β,26-diHC and 26H,7O-C were interconvertible by the same enzymes in the same manner (Fig. 10). HSD11B2 was shown to provide a route to the deactivation of the RORγt agonist 7β,26-diHC, and this was confirmed in a RORγ reporter system where HSD11B2-mediated oxidation of 7β,26-diHC led reduced activation of the receptor (159). Beck *et al.* (159) also showed that CYP27A1 can convert 7β-HC to 7β,26-diHC, whereas earlier studies have shown that 7-OC can be metabolized to 26H,7O-C by CYP27A1 (160). Beck *et al.* (159) did not investigate the alternative deactivation route of 7β,26-diHC to the cholestenoic acid 3β,7β-diHCA, although the presence of this acid in plasma from patients suffering from Smith-Lemli-Opitz syndrome and NPC1 disease and also healthy people suggests that this reaction proceeds in human (95, 113, 155).

These mechanisms for deactivation of 7β,26-diHC are also relevant to its potential role as a ligand toward GPR183. In this context, Beck *et al.* (161) in a separate study demonstrated that 7β,25-diHC, which like 7β,26-diHC, is a ligand toward GPR183, is oxidized by HSD11B2 to inactive 25-hydroxy-7-oxocholesterol (25H,7O-C), which can be reduced back to 7β,25-diHC by HSD11B1. In this study, Beck *et al.* (161) also showed that 7-OC could be hydroxylated by CH25H to 25H,7-OC, providing a further route to GPR183 ligands this time from 7-OC by CH25H-mediated 25-hydroxylation followed by HSD11B1-catalyzed reduction of the 7-oxo group to a 7β-hydroxy group.

Besides oxysterols, it should be noted that cholesterol precursors including desmosterol and 4,4-disubstituted sterols have been proposed as mammalian ligands to RORγt (162, 163).

## Final considerations

In this review, we have tried to demonstrate the importance of oxysterols in the immune response. We have discussed in some details specific articles that we find to be particularly significant and interesting. During our preparation for this review, a number of thoughts relating to experimental conditions came to mind and are worthy of brief discussion here, as are considerations relating to the interpretation of data.

### Replete or delipidated medium, vehicle, esterified or nonesterified sterols

During in vitro experiments, how does the nature of the cell culture medium influence the result? Very often, studies of oxysterol activity are performed in lipid-depleted medium; this is to avoid the uncontrolled addition of oxysterols to cells. However, under these conditions, receptor-mediated endocytosis is not active, and the SREBP-2 pathway is turned on providing the cell with cholesterol exclusively via this route. Under these conditions, cholesterol is not in excess, and oxysterol synthesis in the cell will be restricted. Alternatively, under conditions where the medium is not lipid depleted, receptor-mediated cholesterol uptake is active leading to the plasma membrane being replete with accessible cholesterol, and the excess becomes available for oxysterol formation. This leads to the unanswered question: does oxysterol synthesis depends on the source of the cholesterol substrate? Another aspect to be considered is the vehicle in which the oxysterol is added to cells. A vehicle is necessary on account of the low solubility of oxysterols in aqueous media. Very often, ethanol or methanol is used as the vehicle, and the free sterol is assumed to cross the plasma membrane. Care must be taken to maintain sufficient solubility of oxysterol but to avoid any effects specific to the vehicle itself. But how relevant is the use of a vehicle? The majority of oxysterols are present in the circulation in their esterified forms, so if oxysterol action is considered as hormonal, or prohormonal, then it may be more appropriate to add the esterified oxysterol to the cell in lipoprotein particles. This leads to the next question, when oxysterol data are presented, do the data refer to the free unesterified oxysterol or to the combination of esterified and nonesterified molecules determined following base hydrolysis? The analytical chemist will be able in most cases to answer this question by tracing the experimental method, but this may not be the case for the biologist who may easily be misled unless data are clearly represented. The value for an oxysterol concentration determined following base hydrolysis can be as much as 10-fold greater than for the nonesterified molecule. In this review, values given are those of the unesterified molecules unless stated otherwise.

### Nomenclature, isomers, mouse, and human

The nomenclature resulting from substitution at the terminal carbons of the cholesterol side chain can be confusing (28). For example, the product of the CYP27A1-catalyzed hydroxylation of cholesterol is according to systematic rules 26-HC, [(25R)26-HC], although it is most commonly referred to as 27-HC. Similarly, 7α-hydroxylation of (25R)26-HC by CYP7B1 leads 7α,(25R)26-dihydroxycholesterol, which again is often referred to by the nonsystematic name 7α,27-dihydroxycholesterol. Confusion is further heightened as 7α,26-diHC is found in man and mouse as both the 25R-epimer and 25S-epimer (113, 132, 133) and rarely is the stereochemistry at C-25 defined although the isomers are diastereomeric and can be chromatographically resolved (113).

A further point of consideration is that many results are presented, and conclusions drawn, from experiments made on knockout mice. It is important to realize that while the basic sterol biochemistry in mouse and human is generally similar, as are oxysterol patterns, the phenotype of knockout animals can be very different from the disease symptoms resulting from the corresponding enzyme deficiencies in human. This is especially evident in the case of deficiency in the enzymes CYP7B1 and CYP27A1.

In a similar context, an early report of *Ch25h* expression in mouse indicated *low* levels in heart, lung, and kidney, whereas in human, *CH25H* was more generally expressed but at *very low* levels (15). More recent reports indicate *Ch25h* to be more ubiquitously expressed in mouse than first described (164), whereas *CH25H* was found to be enhanced in brain and lymphoid tissue of human (165). It is difficult to extrapolate to protein expression and whether it is greater in mouse than man or vice versa, but the levels of nonesterified 25-HC in mouse and human plasma are not drastically different at about 1 and 1–5 ng/ml, respectively (31, 113, 132, 166), which would imply that at least in the absence of infection, CH25H protein levels are quite similar. This is backed up by 25-HC levels in plasma from *Cyp7b1*^−/−^ mice and SPG5 patients being at 40–50 ng/ml in both species (31, 166).

## Conclusion

Our understanding of the involvement of oxysterols in immunology was revolutionized with the discovery that *Ch25h* is an IFN-stimulated gene and that macrophages synthesize 25-HC in response to activation of TLRs. The related discovery that 25-HC is generated by macrophages in response to viral and bacterial infections and that 25-HC is protective against both viral and bacterial spread further highlighted the importance of this family of sterols in immunology. The involvement of 7-hydroxylated derivatives of both 25-HC and 26-HC in the adaptive immune response acting as chemoattractants to GPR183-expressing immune cells and of 7α/β,26-diHC epimers as ligands to RORγt has opened more avenues for investigation, particularly with respect to autoimmune diseases. Interestingly, it appears that while 25-HC and its 7-hydroxy metabolites are synthesized specifically in response to infection, 26-HC and its metabolites, which have similar biological activity to those of 25-HC but are continuously present under steady-state conditions, may play a more protective role against infection.

## Data availability

All data are contained within the article.

## Supplemental data

This article contains supplemental data.

## Conflict of interest

W. J. G. and Y. W. are listed as inventors on the patent “Kit and method for quantitative detection of steroids” (US9851368B2). W. J. G. and Y. W. are shareholders in CholesteniX Ltd.
