# Supplementary material for: Cholesterol metabolism: from lipidomics to immunology
Source: J Lipid Res. 2021 Dec 22;63(2):100165. doi: 10.1016/j.jlr.2021.100165 (PMC8953665; doi:10.1016/j.jlr.2021.100165)
Supplement: Supplemental Figures S1–S3 [file mmc1.pptx]

## Slide 1
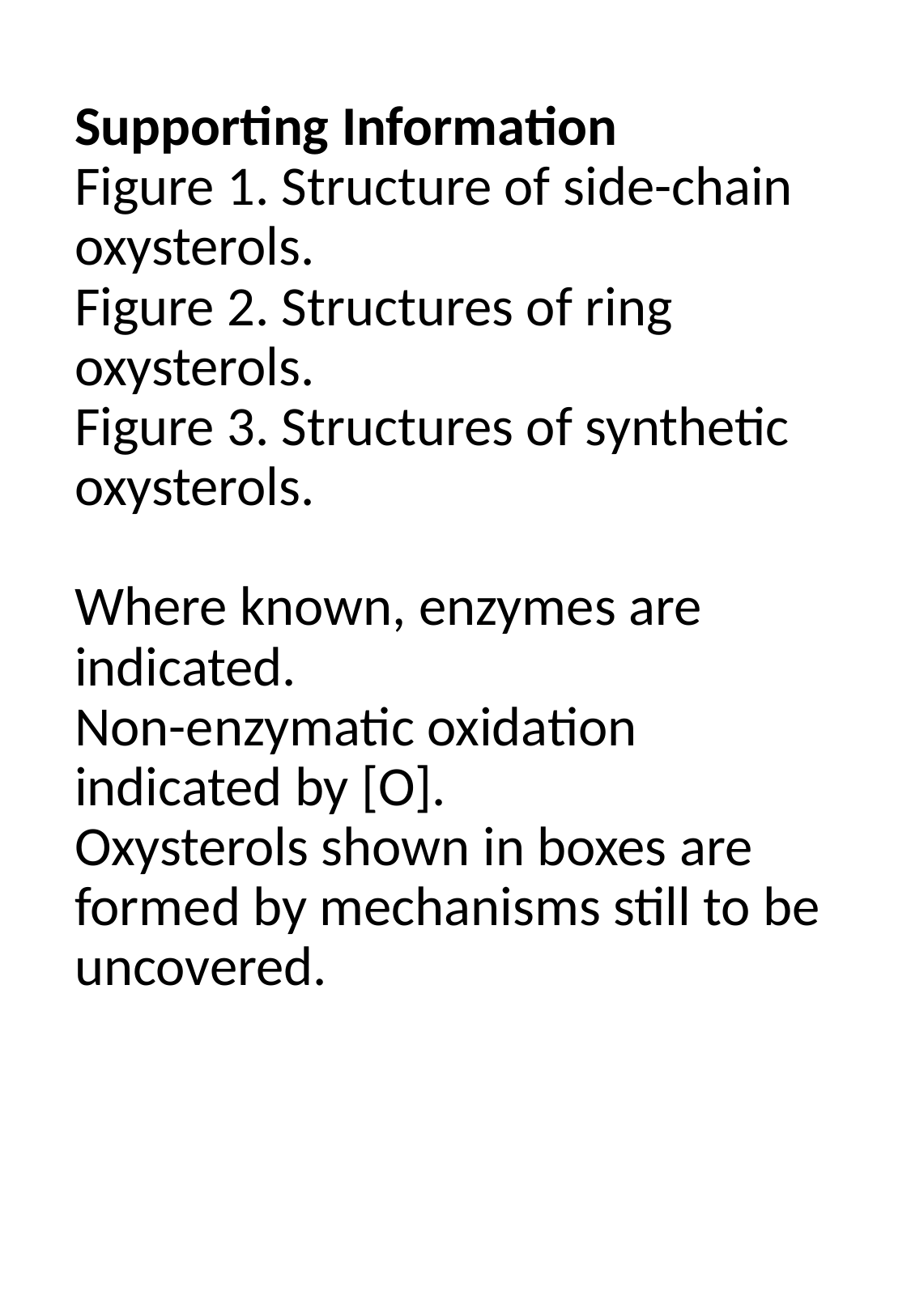

# Supporting Information Figure 1. Structure of side-chain oxysterols. Figure 2. Structures of ring oxysterols. Figure 3. Structures of synthetic oxysterols. Where known, enzymes are indicated. Non-enzymatic oxidation indicated by [O]. Oxysterols shown in boxes are formed by mechanisms still to be uncovered.

## Slide 2
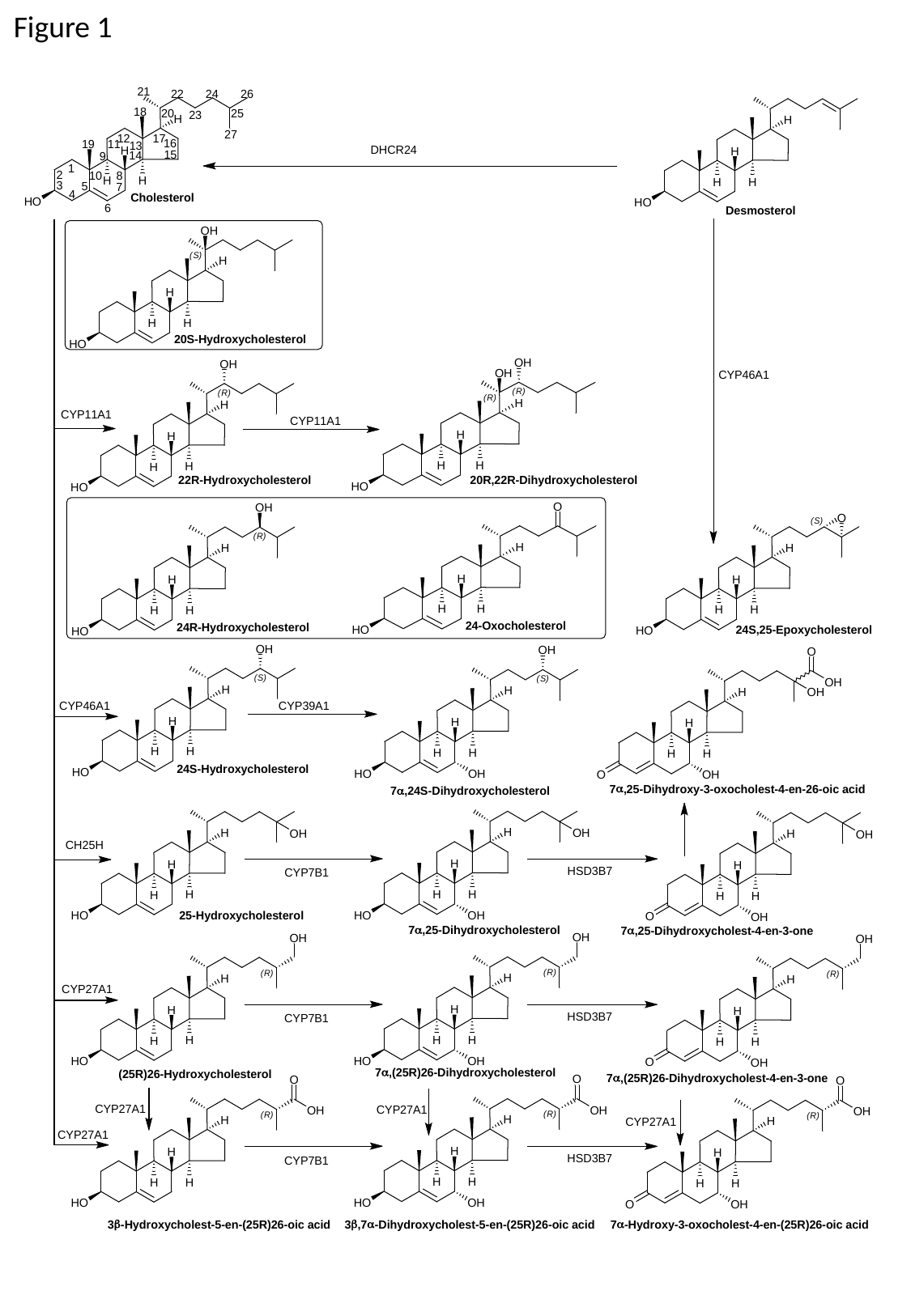

Figure 1

## Slide 3
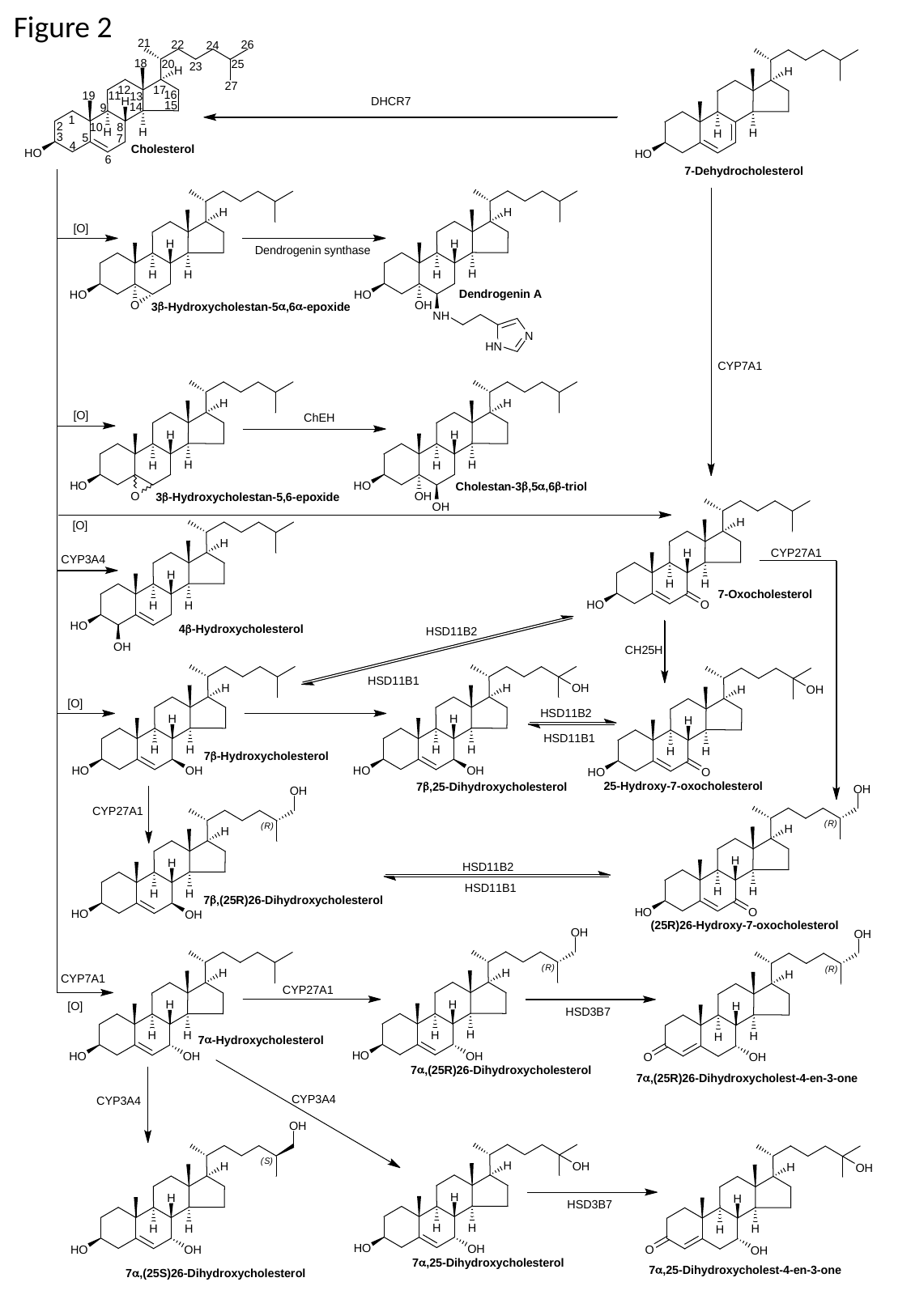

Figure 2

## Slide 4
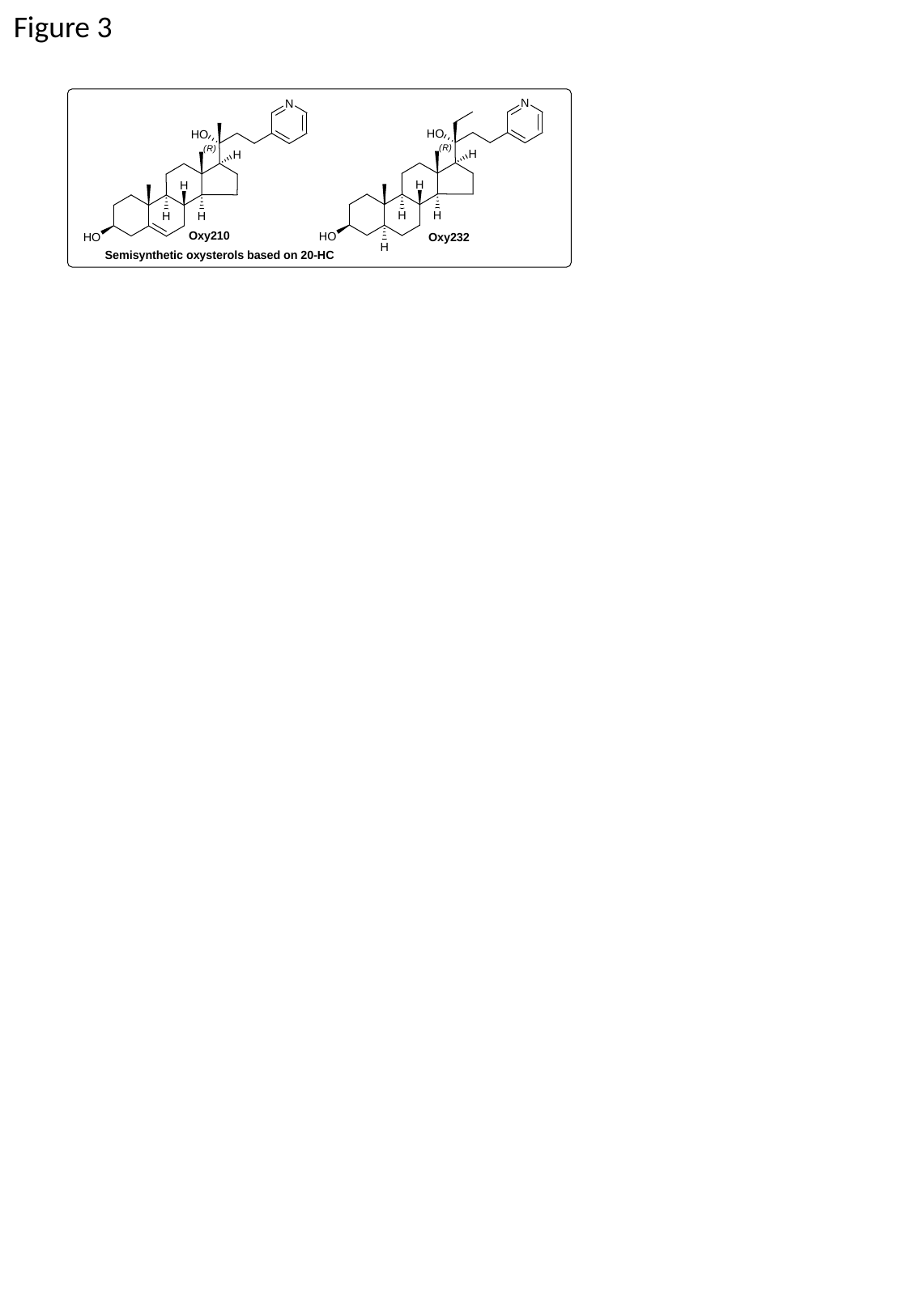

Figure 3
